# Cooperative Transcription Factor Induction Mediates Hemogenic Reprogramming

**DOI:** 10.1016/j.celrep.2018.11.032

**Published:** 2018-12-04

**Authors:** Andreia M. Gomes, Ilia Kurochkin, Betty Chang, Michael Daniel, Kenneth Law, Namita Satija, Alexander Lachmann, Zichen Wang, Lino Ferreira, Avi Ma’ayan, Benjamin K. Chen, Dmitri Papatsenko, Ihor R. Lemischka, Kateri A. Moore, Carlos-Filipe Pereira

**Affiliations:** 1 Department of Cell, Developmental and Regenerative Biology, Icahn School of Medicine at Mount Sinai, One Gustave L. Levy Place, Box 1496, New York, NY 10029, USA; 2 Department of Pharmacological Sciences, Icahn School of Medicine at Mount Sinai, One Gustave L. Levy Place, Box 1496, New York, NY 10029, USA; 3 Black Family Stem Cell Institute, Icahn School of Medicine at Mount Sinai, One Gustave L. Levy Place, Box 1496, New York, NY 10029, USA; 4 Division of Infectious Disease, Department of Medicine, Immunology Institute, Icahn School of Medicine at Mount Sinai, One Gustave L. Levy Place, Box 1496, New York, NY 10029, USA; 5 The Graduate School of Biomedical Sciences, Icahn School of Medicine at Mount Sinai, One Gustave L. Levy Place, Box 1496, New York, NY 10029, USA; 6 Skolkovo Institute of Science and Technology, Nobel Street, Building 3, Moscow 143026, Russia; 7 Doctoral Programme in Experimental Biology and Biomedicine, University of Coimbra, Largo Marquês do Pombal 3004-517, Coimbra, Portugal; 8 Centre for Neuroscience and Cell Biology, University of Coimbra, Largo Marqueŝ do Pombal 3004-517, Coimbra, Portugal; 9 Molecular Medicine and Gene Therapy, Lund Stem Cell Centre, Lund University, BMC A12, 221 84, Lund, Sweden; 10 Wallenberg Centre for Molecular Medicine, Lund University, Lund, Sweden; 11 Senior author; 12 These authors contributed equally; 13 Lead Contact

## Abstract

During development, hematopoietic stem and progenitor cells (HSPCs) arise from specialized endothelial cells by a process termed endothelial-to-hematopoietic transition (EHT). The genetic program driving human HSPC emergence remains largely unknown. We previously reported that the generation of hemogenic precursor cells from mouse fibroblasts recapitulates developmental hematopoiesis. Here, we demonstrate that human fibroblasts can be reprogrammed into hemogenic cells by the same transcription factors. Induced cells display dynamic EHT transcriptional programs, generate hematopoietic progeny, possess HSPC cell surface phenotype, and repopulate immunodeficient mice for 3 months. Mechanistically, GATA2 and GFI1B interact and co-occupy a cohort of targets. This cooperative binding is reflected by engagement of open enhancers and promoters, initiating silencing of fibroblast genes and activating the hemogenic program. However, GATA2 displays dominant and independent targeting activity during the early phases of reprogramming. These findings shed light on the processes controlling human HSC specification and support generation of reprogrammed HSCs for clinical applications.

## In Brief

Gomes et al. show that specification of hemogenesis in human fibroblasts is mediated by cooperative transcription factor binding. GATA2 displays dominance, interacts with GFI1B, and recruits FOS to open chromatin, simultaneously silencing the fibroblast program and initiating an endothelial-to-hematopoietic transition to definitive hematopoiesis.

## Graphical Abstract

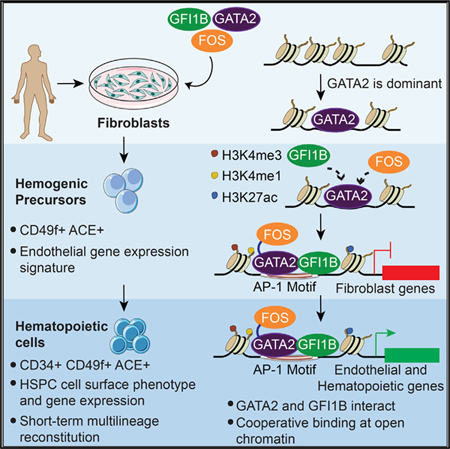

## INTRODUCTION

Early human blood development occurs through sequential stages in which transient hematopoietic cells support the embryo, followed by the emergence of the first hematopoietic stem cells (HSCs). HSCs are generated in the dorsal aorta of the aorta-gonad-mesonephros (AGM) region, subsequently migrate to the fetal liver, and lodge in the adult bone marrow (Ivanovs et al., 2011, 2017; Tavian et al., 2010). In addition, the placenta was identified as a site for human HSC development (Muench et al., 2017; Robin et al., 2009). Human HSCs develop from an intermediate hemogenic precursor cell with endothelial properties between days 27 and 40 (Ivanovs et al., 2017; Oberlin et al., 2002). Evidence from several non-human experimental models suggests that endothelial-to-hematopoietic transition (EHT) is a conserved developmental process (Medvinsky et al., 2011). Human HSCs bud predominantly from the endothelial floor of the dorsal aorta, co-express endothelial and hematopoietic markers, and together with non-self-renewing hematopoietic progenitors form the intra-aortic hematopoietic clusters (Tavian et al., 2010). Although there is no established phenotype that discriminates emergent human HSCs from their precursors or progenitors, some molecules have been identified that are present in developing HSCs. Angiotensin-converting enzyme (ACE) marks fetal liver HSCs (Jokubaitis et al., 2008) and ACE+CD34− cells beneath the human dorsal aorta (Sinka et al., 2012). ACE+CD34− cells may represent HSC precursors that give rise to ACE+CD34+ cells contained in aortic clusters. Human long-term repopulating HSCs reside in the CD34+CD38^low^CD90+ population of umbilical cord blood (UCB) (Majeti et al., 2007). Further studies have shown that integrin alpha 6 (CD49f) (Notta et al., 2011) in UCB and GPI-80 (Prashad et al., 2015) in fetal liver further purifies self-renewing HSCs.

Directed differentiation of human pluripotent stem cells (PSCs) has provided valuable information about the transcriptional program of hematopoiesis. Human PSC-derived hemogenic cells are distinguished by expression of the transcription factors (TFs) RUNX1, GFI1, and GFI1B, which are essential for EHT (Ng et al., 2016). Recent studies have shown that SOX17-positive endothelial cells are generated during PSC differentiation and subsequently activate RUNX1 during EHT (Ng et al., 2016). Thus far, current protocols for hematopoietic differentiation of human PSCs remain skewed toward extra-embryonic hematopoiesis rather than intra-embryonic definitive HSC formation (Ditadi et al., 2017; Ng et al., 2016). TFs crucially important for hematopoietic development including GATA2 and RUNX1 are up-regulated in human intra-aortic clusters (Labastie et al., 1998). How these regulators promote definitive human hematopoiesis is unknown. Putative mechanisms include pioneer hematopoietic TFs that bind and prime closed chromatin (Soufi et al., 2012; Wapinski et al., 2013) or TFs that interact and cooperatively engage open chromatin (Chronis et al., 2017). Studies in mouse HSPCs have shown combinatorial interaction between a heptad of TFs (SCL, LYL1, LMO2, GATA2, RUNX1, ERG, and FLI-1) in hematopoietic progenitors (Wilson et al., 2010). During mouse PSC differentiation the cooperative binding of AP-1 with TEAD4 was shown to promote a hemogenic cell fate at the expense of alternative cell fates (Obier et al., 2016). As hematopoietic progenitors differentiate, GATA2 binding persists in erythroid cells, acting as a ‘‘pioneer’’ or nucleation factor for the recruitment of GATA1, indicating that GATA2 and GATA1 cooperate extensively to regulate erythroid differentiation (May et al., 2013). Difficult availability of material hinders a detailed understanding of the transcriptional control of human HSC specification.

We have previously shown the direct reprogramming of mouse fibroblasts into hemogenic precursors cells using GATA2, FOS and GFI1B with increased efficiency with ETV6 (Pereira et al., 2013). Induction leads to a dynamic process that progresses through an endothelial-like intermediate with a defined phenotype (Prom1+Sca-1+CD34+CD45−). Using this phenotype, we identified a population *in vivo* that expresses endothelial and early hematopoietic markers, localizes in the vascular labyrinth of mouse placenta, and upon co-culture with stromal cells will engraft primary and secondary mice (Pereira et al., 2016). Therefore, mouse hemogenic reprogramming recapitulates developmental hematopoiesis. Recently it was demonstrated that the expression of GATA2, FOS, and GFI1B within PSC-derived teratomas leads to the generation of long-term repopulating HSCs (Tsukada et al., 2017).

Here, we show that GATA2, GFI1B, and FOS reprogram human fibroblasts into hematopoietic progenitors that transit through an intermediate with endothelial properties. These cells acquire emergent HSPC-like gene expression profiles and cell surface phenotypes and will repopulate NOD-*scid IL2R*g-*null* (NSG) mice. We have established that collaborative interactions among the three TFs engage open enhancers and promoters that mediate both the silencing of fibroblast-specific genes and activation of endothelial and hematopoietic genes. This molecular analysis of TF targeting provides insight into the regulatory machinery required for hematopoietic reprogramming and reveals the importance of TF cooperation during HSC specification.

## RESULTS

### Transferring Hemogenic Reprogramming to the Human System

**Figure 1:**
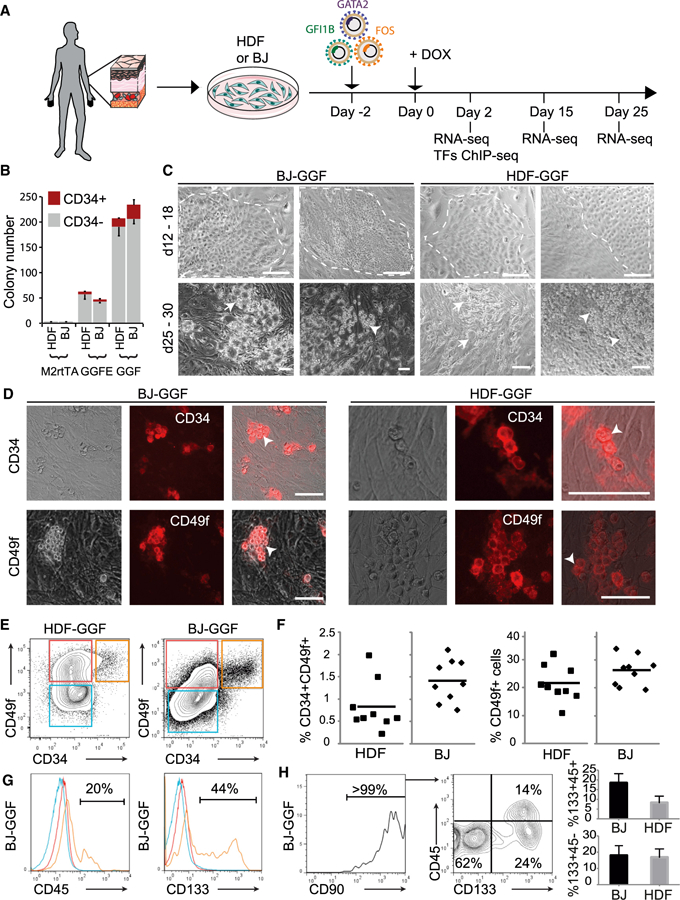
GATA2, GFI1B, and FOS Induce CD34+ and CD49f+ Colonies in Human fibroblasts (A) Strategy for inducing hemogenesis in human dermal fibroblasts (HDFs) and neonatal foreskin fibroblasts (BJ). (B) Cells were transduced with GATA2, GFI1B, FOS, and ETV6 (GGFE); GATA2, GFI1B, and FOS (GGF); or control M2rtTA viruses and cultured with Dox for 30 days. Colonies were stained for CD34 and counted. Colony numbers are per 10,000 transduced fibroblasts (mean ± SD, n = 3). (C) Colony morphology 12–18 days (top, dashed line) and 25–30 days (bottom) after induction. Arrows highlight endothelial and arrowheads highlight induced hematopoietic cellular morphologies. (D) Colonies were assayed by immunofluorescence for CD34 (top) or CD49f (bottom) 30 days after transduction. Arrowheads highlight induced hematopoietic morphologies. Scale bars, 100 μm. (E) Analysis of CD34 and CD49f expression 26 days after transduction with GGF. (F) Quantification of CD34+CD49f+ and CD49f+ cell populations. Each symbol represents an experiment and the horizontal bar indicates the mean. (G) Expression of CD45 and CD133 within CD34+CD49f+ (orange lines), CD49f+ (red lines), and double-negative population (blue lines). (H) Expression of CD90, CD45, and CD133 within the CD34+CD49f+ population. Quantification of the CD133+CD45− (bottom) and CD133+CD45+ (top) populations from three biological replicates between days 23 and 26 (mean ± SD, n = 3). See also Figures S1 and S2.

To assess the feasibility of using the same TFs used in the mouse, we determined their combinatorial expression in human cells with in-house-developed software, GPSforGenes. We found that both GGF (GATA2, GFI1B, and FOS) and GGFE (GATA2, GFI1B, FOS, and ETV6) combinations were highly expressed in human CD34+ hematopoietic stem and progenitor cells (HSPCs) (Figures S1A and S1B). We next expressed the human TFs in adult human dermal fibroblasts (HDFs) and neonatal foreskin (BJ) fibroblasts using a doxycycline (Dox)-inducible vector system (Pereira et al., 2013). Two days after transduction with GGFE and GGF viruses, Dox was added and cultures were analyzed for more than 25 days (Figure 1A). Interestingly, in human cells the GGF minimal cocktail appears more effective than GGFE (Figure 1B). The colony formation efficiency ranged from 2.1 ± 0.2% to 2.3 ± 0.1% with GGF and from 0.4 ± 0.1% to 0.6 ± 0.1% with GGFE. An analysis of both colony number and expression of CD34 revealed that 8.4%–13.6% of the colonies contained CD34+ cells (Figure 1B). Similar morphological changes were observed in both HDF and BJ transduced cultures. The cells appeared to round up in cobblestone-like areas during the midpoint of the culture with semi-adherent and non-adherent round cells appearing later (Figure 1C). Round hematopoietic-like cells that express CD34 and CD49f by immunofluorescence were observed (Figure 1D) and further quantified by flow cytometry (Figure S1C). These changes defined the induction period; a large population of cells expressed CD49f+ (20%–25%), while CD34 expression was more restricted (0.2%–2.0%). The CD49f+ population emerges early (day 12) and is maintained, while CD34+ cells peak at day 25 and subsequently decline but do persist. No colonies, morphologic changes, or CD49f+ and CD34+ cells were observed after transduction with control viruses (Figures S1D and S1E).

### Induced Colonies Contain Cells with Human HSPC Surface Phenotypes

We examined the induced colonies for co-expression of markers that define a human HSC phenotype. Both HDF and BJ derived cells co-express CD34 and CD49f (Figure 1E). Transduced BJ cells generated greater numbers of CD49f+ (positive for CD49f and CD34-negative) and CD34+CD49f+ cells (Figure 1F), possibly because of their neonatal origin. A subpopulation of CD34+CD49f+ cells expressed CD133 (Prominin1) and low levels of CD45, which is expressed at low levels in UCB HSCs (Figures 1G, 1H, and S2A) (Jay et al., 2004). CD34+CD49f+ cells also express CD90 (Thy1) (Figures 1H and S2B). The negative markers CD38 and CD45RA were not detected (Figure S2C). These data correlate with extensive phenotypic definitions of human HSCs in UCB (Notta et al., 2011). Interestingly, GATA2 or FOS removal from the three-factor combination abolished the generation of CD34+CD49f+ cells, while removal of GFI1B increased the number of double-positive cells (Figure S2D) but diminished the percentage of CD45+ and CD133+ cells (Figure S2E), consistent with the role of GFI1B during mouse EHT (Pereira et al., 2016; Thambyrajah et al., 2016). These results demonstrate that GGF induce a human HSPC phenotype in two different types of human fibroblasts.

### Comprehensive Gene Expression Analyses during Reprogramming

**Figure 2:**
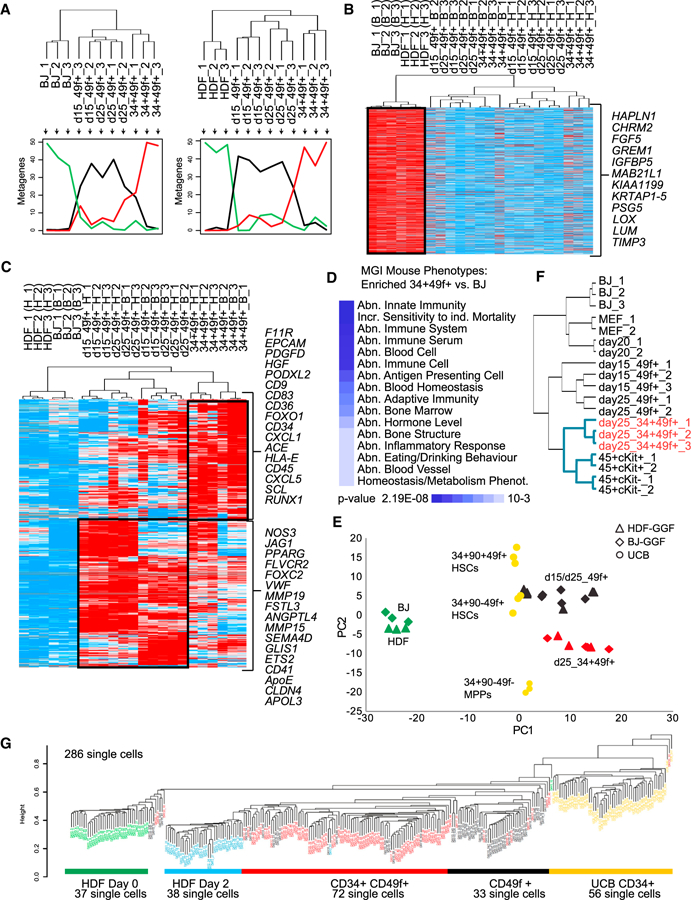
Dynamic Activation of Endothelial and HSPC-like Gene Expression Signatures in Reprogrammed Cells (A) Populations of non-transduced fibroblasts and GGF transduced day 15 CD49f+, day 25 CD49f+, and day 25 CD34+CD49f+ cells were profiled using RNA-seq (three biological replicates; samples that did not pass quality control were discarded). Ordered tree linkage displays clustering of the profiled samples and the metagenes that represent most of the variability associated with each cellular transition. (B) Heatmap of genes expressed in fibroblasts and silenced in CD49f+ and CD34+CD49f+ cells. (C) Heatmap of genes activated in CD49f+ and CD34+CD49f+ cells. Black boxes highlight the stage-specific expression of gene sets. Red indicates increased expression and blue decreased expression over the mean. Data were analyzed using Cluster 3.0 and displayed using Treeview. (D) Gene list enrichment analysis with libraries from MGI mutant mouse phenotype ontology for genes upregulated from BJ to CD34+CD49f+. Heatmap shows enrichment p values. (E) RNA-seq datasets were integrated with expression data from human UCB hematopoietic stem and progenitor cells (HSPCs) (from Notta et al., 2011). PCA shows the relative distances between samples. (F) Hierarchical clustering integrating data from reprogrammed mouse and human cells (mouse data from Pereira et al., 2013); human CD34+CD49f+ cells highlighted in red. Blue lines highlight separate cluster for hematopoietic phenotypes. (G) Non-supervised hierarchical clustering showing genome-wide gene expression data from HDF-derived single cells. UCB CD34+ single cells are marked in yellow. The number of single cells analyzed and phenotype are detailed. See also Figures S3 and S4 and Tables S1, S2, and S4.

We interrogated the gene expression changes occurring during reprogramming by mRNA sequencing (RNA-seq) analysis of cell populations. We sorted three biological replicates of non-transduced fibroblasts, day 15 CD49f+, day 25 CD49f+, and CD34+CD49f+ cells from both types of fibroblasts (Figure 1A; Table S1). Biological replicates correlated well, and metagene analyses that represent most of the variability associated with BJ and HDF reprogramming showed (1) sets of genes expressed in fibroblasts and silenced in all other samples (Figure 2A, green lines), (2) genes transiently expressed in CD49f+ cells (Figure 2A, black lines), and (3) genes that start to be expressed in CD34+CD49f+ cells (Figure 2A, red lines). Examples of sequential changes include *MMP1*, a fibroblast-associated gene silenced in sorted populations; *ANGPTL4*, implicated in the positive regulation of angiogenesis, transiently activated in CD49f+ cells; and *ACE*, upregulated in CD49f+ cells with continued expression in CD34+CD49f+ cells (Figures S3A). We confirmed ACE and CD49f expression in transduced cells and found that CD34+ cells are contained within this subset (Figure S3B). Integration of the RNA-seq datasets revealed silencing of the fibroblast-associated gene signature (Yu et al., 2007), which includes *GREM1*, *PSG5*, *FGF5*, *LUM*, *HAPLN1*, and *MAB21L* (Figures 2B and S3C). Among upregulated genes in CD34+CD49f+ cells we identified proposed markers of AGM HSC precursors such as *ACE*, *F11R*, and *EPCAM*, the TFs *RUNX1*, *SCL*, and *FOXO1*, and the *CD9* homing molecule. Several proangiogenic genes (*JAG1*, *SEMA4D*, *VWF*, *FOXC2*, and *ETS2*) are expressed in CD49f+ cells at both days 15 and 25 (Figures 2C and S3C). We asked if an angiogenic program is activated during reprogramming from a list of positive angiogenic regulators. The vast majority of these genes are activated in CD49f+ cells, and some continue to be expressed in CD34+CD49f+ cells (Figures S4A and S4B). Gene set enrichment analysis (GSEA) revealed enrichment for and regulation of angiogenesis (Figure S4C). Further analyses of genes upregulated in CD34+CD49f+ cells using the Mouse Genome Informatics (MGI) mouse mutant phenotype database showed that their genetic perturbations result in largely hematopoietic phenotypes (Figure 2D). We used GSEA to compare the transition from fibroblasts to CD34+CD49f+ cells. A significant number of HSPC gene sets were enriched (31 and 26 enriched versus 1 and 6 non-enriched gene sets) in CD34+CD49f+ cells generated from either type of fibroblasts (Figure S4D, left). Indeed, top enriched gene sets include CD34 TFs as well as the Wnt, Hedgehog, and TGFβ signaling pathways, consistent with their hemogenic roles (Figure S4D, right) (Medvinsky et al., 2011; Pereira et al., 2016). We next integrated our data with published HSC data from UCB (Notta et al., 2011). Principal-component analysis (PCA) showed that induced CD49f+ cells are the closest to CD49f+ HSCs, while both HDF and BJ fibroblasts are very distinct from all other datasets. The CD34+CD49f+ cell populations are positioned between HSCs and multipotent progenitors (MPPs) (Figure 2E). A comparison of our mouse reprogramming data (Pereira et al., 2013) with the human showed that CD34+CD49f+ cells cluster closely with mouse induced HSPCs (CD45+cKit+) (Figure 2F). To characterize the reprogramming process in more detail, we performed single-cell RNA-seq throughout induction. UCB Lin-CD34+ cells and non-transduced HDFs were profiled as controls. Genome-wide unsupervised hierarchical clustering shows that most single cells cluster according to sample group, with a clear separation of HDF from reprogrammed cells. Just 2 days after transgene activation, fibroblasts show a dramatic transcriptional change. Importantly, endogenous expression of *GFI1B* and *GATA2* is absent at day 2 but activated in reprogrammed cells (Figure S4F), suggesting that the reprogramming process is stable and becomes independent of exogenous expression of GFI1B and GATA2. *FOS* is expressed in fibroblasts and continues to be expressed in reprogrammed cells. As in the PCA (Figure 2E), single CD49f+ cells are the closest to human UCB cells (Figure 2G). These data suggest that, with time in culture, these cells acquire the expression of differentiation-related genes, along with CD34. We performed GSEA with gene lists for HSCs and MPPs (Notta et al., 2011) on single-cell mRNA-seq data (Figure S4G). MPP genes are significantly enriched in the CD34+CD49f+ subset when compared to CD49f+. For the HSC gene set there is no clear enrichment in this transition, indicating that those genes are already expressed in the CD49f+ population. To provide additional information on genes that may be missing from reprogrammed CD49f+ cells, we analyzed the expression of the HSC signature genes (Notta et al., 2011) individually. We have detected 29 of 39 genes. From the 10 genes that were not detected in reprogrammed cells, only 1 (*SOX18*) is a TF (Table S2). In terms of signaling, pathways such as Notch and integrin alpha 6 are enriched in CD49f+ cells, while TGFβ, WNT, and T cell receptor signaling are enriched in CD34+CD49f+ (Figure S4H). To generate information on the genes and pathways changing at each transition, we performed gene list enrichment analysis (Enrichr) (Chen et al., 2013) for all possible pairwise comparisons in the single-cell data. An interactive comparative map was created using the 500 most differentially expressed genes (Figure S4E; see Table S2 for gene lists). The hyperlinks allow exploration of the data and the identification of ontologies, pathways, diseases and drugs, and so on, implicated in each cellular transition. For example, glycolysis and INFα signaling pathways (p = 2.7 × 10^−5^ and p = 1.2 × 10^−3^, respectively) were the top enriched terms for the transcriptional activation occurring in the first 2 days of reprogramming, suggesting that these genes may be direct targets of hemogenic TFs. Taken together, GGF direct hemogenic transcriptional changes in multiple types of fibroblasts of both mouse and human origins.

### Induced Human Cells Engraft In Vivo

**Figure 3:**
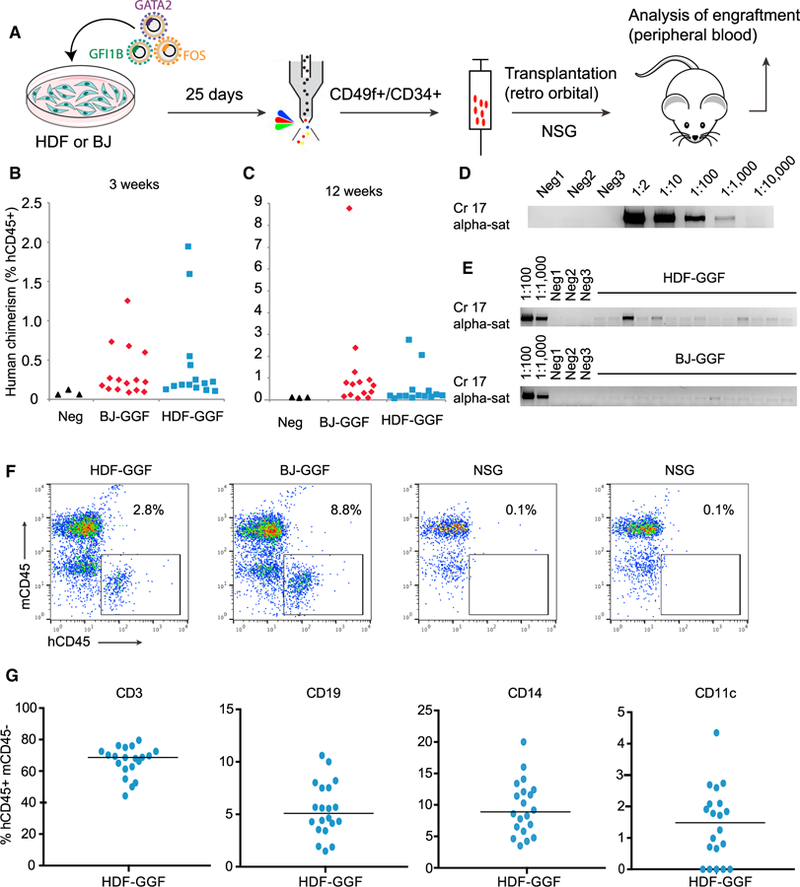
Reprogrammed Cells Engraft *In Vivo* after Transplantation (A) Experimental design used to sort and transplant induced cells into NOD-*scid IL2R*γ-*null* (NSG) mice. HDF or BJ fibroblasts were transduced with GGF and cultured with Dox for 25 days. Cells were dissociated, and CD49f+ (including CD34+CD49f+ double-positive cells) were sorted and then injected into 4-week-old NSG mice. (B) Human chimerism in peripheral blood 3 weeks after transplantation with HDF-GGF (n = 15) or BJ-GGF (n = 14). (C) Percentage of human CD45 chimerism 12 weeks after transplantation. (D) Limit of detection of human engraftment was tested by PCR using human-specific primer pairs (chromosome 17 alpha satellite) from serial dilutions of human HDFs mixed with mouse cells. Mouse cells only were used as negative controls. (E) Human chimerism in peripheral blood was tested using PCR 4 weeks after transplantation. Each lane represents one individual mouse 4 weeks after transplantation with HDF-GGF (top) or BJ-GGF (bottom). Blood from non-transplanted mice was used as negative controls. (F) FACS plots showing individual mice analyzed with human CD45 (hCD45) and mouse CD45 (mCD45) antibodies. (G) Lineage marker analysis in gated hCD45+mCD45− cells. Scatterplots show expression of human lymphoid and myeloid markers. Data from 20 mice transplanted with similar number of reprogrammed cells; the horizontal bar indicates the mean.

To determine if reprogrammed HSPCs were functional, we undertook xenogeneic transplants. Fibroblasts were transduced and cultured for 25 days in the presence of Dox. CD34+CD49f+ cells as well as single-positive populations were sorted and transplanted, and NSG mice were maintained on Dox for 2 weeks (Figure 3A). We detected human chimerism in peripheral blood at both 3 and 12 weeks after transplantation (Figures 3B and 3C). Although all mice had low levels of huCD45+ (0.1%–2%) at 3 weeks, the number of positive mice dropped dramatically by 3 months. We confirmed this result at week 4 by PCR using primers to human chromosome 17-alpha-satellite sequences (Figures 3D and 3E). A clear population of human CD45+ mouse CD45- was detected (Figure 3F) by flow cytometry. The huCD45+ cells contained both lymphoid and myeloid cells (Figure 3G). These results demonstrate that inducible expression of *GATA2*, *GFI1B*, and *FOS* converts human fibroblasts into HSPCs that repopulate for at least 3 months. Because chimerism from reprogrammed HSPCs was not sustained, we believe that it is crucial to define the TF mechanisms underlying human hemogenic induction.

### GATA2 Displays Dominant and Independent Targeting Capacity to Initiate Hemogenic Reprogramming

**Figure 4:**
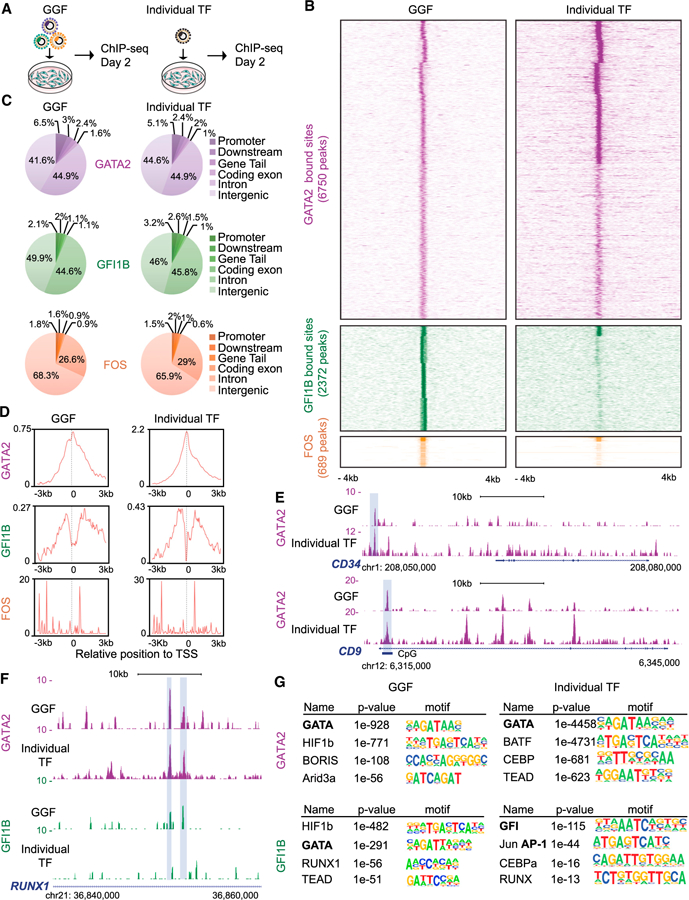
Analysis of TF Occupancy Reveals that GATA2 Has Both Dominant and Independent Targeting Capacity (A) Strategy for identifying GGF genomic binding sites. GGF factors were transduced in combination (left) or individually (right) and analyzed using ChIP-seq 2 days after adding Dox. (B) Heatmaps representing genome-wide occupancy profile for GGF factors when expressed in combination or individually in HDFs. For each site, the signal is displayed within an 8 kb window centered on individual peaks. (C) Genomic distribution of GGF and individual transcription factor (TF) peaks in transduced HDFs. (D) ChIP-seq read density relative to transcription start site (TSS). The graph shows a plot of the average read coverage per million mapped reads centered to the TSS. (E) Genome browser profiles illustrating GATA2-binding sites at *CD34* and *CD9* loci. The y axis represents the total number of mapped reads. The boxes highlight shared peaks when GATA2 is expressed individually or in combination with GFI1B and FOS. (F) GATA2 and GFI1B occupancy profile at the *RUNX1* locus. The boxes highlight shared peaks between GATA2 and GFI1B only when expressed in combination. The genomic scale is in kilobases. (G) *De novo* motif prediction for GATA2 and GFI1B target sites when expressed in combination or individually. The motifs for GATA, GFI, and AP-1 factors are bolded. See also Figure S5 and Table S3.

To define the molecular mechanism underlying hemogenic reprogramming, we determined where the TFs initially bind to the genome. We created tagged versions of the TFs to carry out these experiments (Figure S5A). Western blots and immunofluorescence confirmed expression and nuclear and subnuclear localization, respectively (Figures S5B–S5D) (Vassen et al., 2006). Fibroblasts were transduced with the three factors individually or in combination, after which we performed chromatin immunoprecipitation sequencing (ChIP-seq) (Figure 4A). When the three TFs are expressed in combination, GATA2 showed the most extensive binding to the fibroblast genome (6,750 peaks), followed by GFI1B (2,372 peaks) and FOS (689 peaks) (Figure 4B). Interestingly, GATA2-bound sites were similar when GATA2 was expressed alone or together with GFI1B and FOS, suggesting that GATA2 displays independent targeting capacity. In contrast, GFI1B depends on GATA2 and FOS expression to bind to the majority of its targets. FOS showed a small number of targets, suggesting that it has limited access to chromatin during the initial stages of reprogramming (Figure 4B). GATA2 displayed enrichment at promoters both when is expressed individually (Figure 4C, right) or in combination with GFI1B and FOS (Figure 4C, left) and a greater read density at transcription start sites (TSS) compared with GFI1B and FOS (Figures 4D and S5E), suggesting an important regulatory role. GATA2 targets include the *CD34* gene, the HSC homing molecule *CD9* (Figure 4E) (Karlsson et al., 2013), and the EHT mediator *GPR56* (Figure S5F) (Solaimani Kartalaei et al., 2015). *RUNX1* (Figure 4F) and *BMPER* (Figure S5G) (McGarvey et al., 2017), two important regulators of hematopoiesis, are targeted by both GATA2 and GFI1B in GGF-induced fibroblasts. However, GFI1B binding was lost when GATA2 was not included, suggesting cooperative binding between GATA2 and GFI1B for the induction of *RUNX1* and definitive hematopoiesis. Motif prediction for GATA2 in GGF-induced fibroblasts showed that the GATA motif was strongly enriched. This analysis also identified HIF1B, BORIS, and ARID3A as regulators of hematopoietic reprogramming (Figure 4G, top). For GFI1B targets, AP-1, HIF1B, and GATA motifs were identified, but the GFI motif was strongly enriched only when GFI1B was expressed individually (Figure 4G, bottom). This suggests that GATA2 recruits GFI1B to its ‘‘natural’’ target sites. Overall these results provide insights as to how these factors engage the fibroblast genome and initiate hemogenic programming by targeting key hematopoietic regulators.

### GATA2 and GFI1B Interact and Share a Cohort of Target Sites

**Figure 5:**
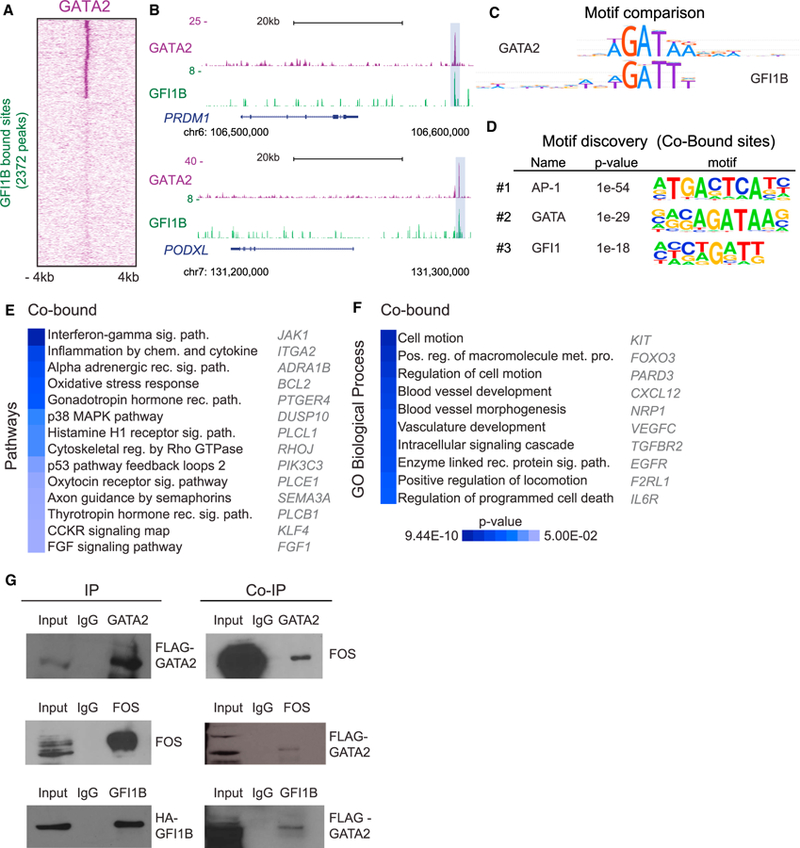
GATA2 and GFI1B Interact and Share a Cohort of Target Sites (A) Heatmap representing genome-wide occupancy profiles for GFI1B showing shared targets with GATA2 48 hr after induction of GGF with Dox. The signal at the corresponding genomic regions of TF binding is displayed across the other dataset. For each site, the signal is displayed within an 8 kb window centered on individual peaks. (B) Genome browser profiles illustrating GATA2- and GFI1B-binding sites at *PRDM1* and *PODXL* loci. The y axis represents the total number of mapped reads. The boxes highlight genomic positions co-occupied by GATA2 and GFI1B. (C) Motif comparison between GATA2 and GFI1B. Jaccard similarity coefficient = 0.1. (D) *De novo* motif discovery at co-bound sites. The top three most enriched motifs are shown along with p values. (E) Panther pathway enrichment analysis of genes co-bound by GATA2 and GFI1B. (F) Biological processes Gene Ontology (GO) terms enriched for co-bound genes. Examples of co-bound genes are shown and heatmaps display p values. (G) Immunoblots showing immunoprecipitation (IP) of GATA2 (top), FOS (middle), and GFI1B (bottom) in HDFs 48 hr after induction of GGF (left). Right: coIP detection for FOS (top) and GATA2 (middle and bottom). Input (10%) indicates non-immunoprecipitated cell lysate, and IgG indicates control IP with isotype antibody. See also Figure S6 for uncropped immunoblots and Table S3.

We next investigated the extent of overlap between GATA2 and GFI1B genomic targets and whether they physically interact. By displaying GATA2 and GFI1B target sites, we observed that 750 genomic positions were shared, representing 31.6% of total GFI1B targets (Figure 5A). These include HSC and EHT regulators such as *PRDM1* and *PODXL* (Figure 5B). Motif comparison analysis showed significant similarity between GATA2 and GFI1B motifs (Jaccard similarity index = 0.1) (Vorontsov et al., 2013), supporting the interaction between the two TFs (Figure 5C). We then performed *de novo* motif prediction for the overlapping peaks. Interestingly, the AP-1 motif was the most enriched, followed by the GATA and GFI1 motifs, highlighting the cooperative action among the three factors during reprogramming (Figure 5D). Co-bound genes are part of pathways such as interferon-gamma signaling, inflammation, and cytoskeletal regulation by Rho GTPases (Figures 5E and S6A), processes with demonstrated relevance for HSC emergence (Pereira et al., 2013, 2016). Gene Ontology analysis of co-bound genes showed that cell motion and vasculature development were enriched terms (Figures 5F and S6B). We further interrogated our ChIP-seq data for the regulatory interactions between the three hemogenic TFs. Both GATA2 and GFI1B bind their own loci at the initial stages of reprogramming, suggesting auto-regulation as previously shown in hematopoietic progenitors (Anguita et al., 2010; May et al., 2013). In addition, GATA2 binds to a CpG island in the *FOS* locus and GFI1B binds to the *GATA2* locus only in the presence of the other two TFs (Figure S6C). We did not detect binding of GATA2 to the *GFI1B* locus, suggesting that this interaction may be established later in hematopoietic progenitors (Moignard et al., 2013). To confirm physical interaction, we have performed co-immunoprecipitation (coIP) 48 hr after expression in fibroblasts. This analysis demonstrated an interaction between GATA2 and FOS and between GATA2 and GFI1B (Figure 5G). This suggests that the interplay among GGF is central for hemogenic reprogramming (Figure S6C).

### GATA2 and GFI1B Engage Open Promoters and Enhancer Regions

**Figure 6:**
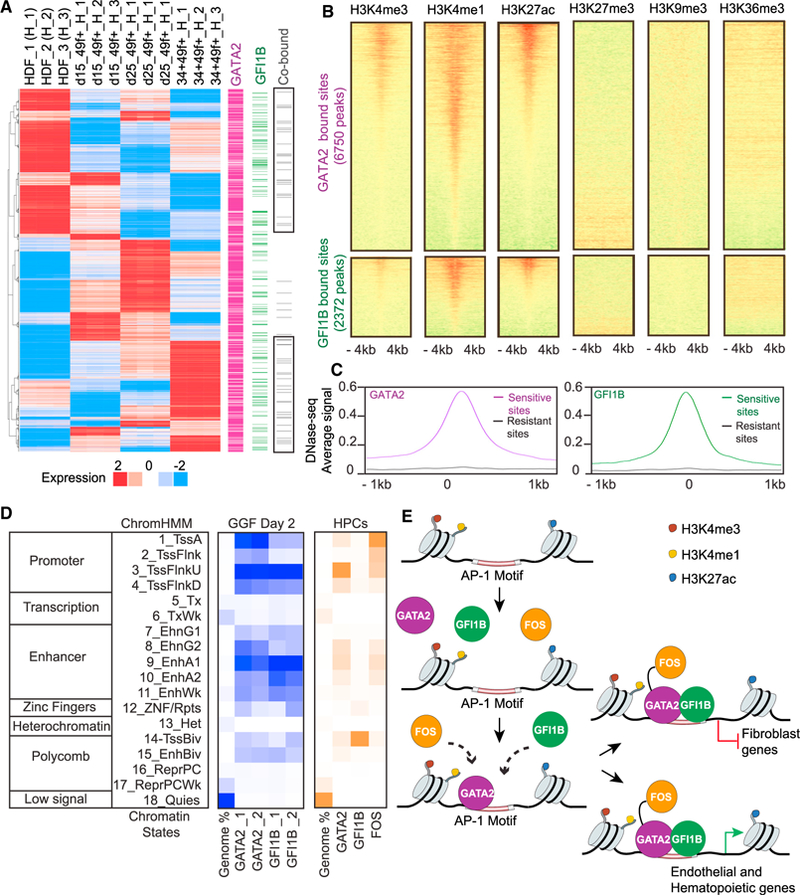
GATA2 and GFI1B Engage Open Promoters and Enhancer Regions (A) Integration of RNA-seq and ChIP-seq datasets. The heatmap (left) shows 1,425 genes with at least 1.5-fold expression changes across the dataset. Genes were identified as targets of GATA2 (purple) or GFI1B (green), with co-bound targets in gray (right), on the basis of binding peaks within the range of a 10 kb window centered on the transcriptional start site. (B) Heatmaps of normalized tag densities representing HDF chromatin marks at GATA2 and GFI1B target sites. The signal is displayed within an 8 kb window centered on the binding sites. (C) Average DNase-seq signal of HDFs at GATA2 and GFI1B target sites. The signal is displayed within a 2 kb window. (D) Heatmaps for chromatin-state functional enrichment. Rows represent chromatin states according to ChromHMM annotation: TssA, active promoters; TssFlnk, flanking promoters; TssFlnkU, flanking upstream promoters; TssFlnkD, flanking downstream promoters; Tx, strong transcription; TxWk, weak transcription; EnhG1/2, genic enhancers; EnhA1/2, active enhances; EnhWk, weak enhancers; ZNF/Rpts, ZNF genes and repeats; Het, heterochromatin; TssBiv, bivalent/poised TSS; EnhBiv, bivalent enhancer; ReprPC, repressed PolyComb; ReprPCWk, weak repressed PolyComb; Quies, quiescent. Blue panel shows the percentage of genome occupancy for GATA2 and GFI1B in GGF-transduced HDFs. Orange panel shows the percentage of genome occupancy for GGF in human hematopoietic progenitor cells (HPCs). (E) Model of the mechanism of action of GGF during hemogenic induction in human fibroblasts. See also Figure S6 and Tables S3 and S4.

We next asked whether GATA2 and/or GFI1B engagement correlates with gene activation or silencing during human reprogramming. We identified 1,425 significantly changing genes (across the population mRNA-seq dataset from HDF-derived cells), which were bound by either GATA2 and/or GFI1B. Specifically, 1,186 genes were bound by GATA2, and 182 were bound only by GFI1B. Fifty-seven differentially expressed genes were co-bound, targeting the cluster of genes highly expressed in fibroblasts and a second cluster of genes enriched only in CD34+CD49f+ cells (Figure 6A; Table S3) (p < 10^−10^, Fisher’s t test). These data suggest that GATA2 and GFI1B co-binding is involved both in the repression of fibroblast-associated genes and activation of hematopoietic-associated genes. To characterize the chromatin features associated with GATA2 and GFI1B engagement, we used previously published ChIP-seq datasets for H3K4me1, H3K4me3, H3K27ac, H3K27me3, H3K9me3, and H3K36me3 in HDFs (Table S4). GATA2- and GFI1B-bound sites in fibroblasts are enriched for marks associated with active promoters and enhancers such as H3K4me3, H3K27ac and H3K4me1 (Figure 6B). This result is consistent with the DNase I accessibility in HDFs. GATA2 and GFI1B bind mostly to DNase I-sensitive sites (Figure 6C; Table S4). These results demonstrate that GATA2 and GFI1B preferentially bind to accessible chromatin primarily in promoter and enhancer regions. We summarized the association between GATA2 and GFI1B binding and chromatin in fibroblasts using ChromHMM, a segmentation of the genome into 18 chromatin states on the basis of the combinatorial patterns of chromatin marks. We confirmed the preference of GATA2 and GFI1B in active TSS, flanking upstream TSS and active enhancers (Figure 6D, blue). In addition, we analyzed published datasets for histone marks in K562 cells (Figure S6E) and GGF TF occupancy in hematopoietic progenitor cells (HPCs) (Table S4). In contrast to GATA2 and FOS, we observed a distinct pattern for GFI1B that is strongly enriched in bivalent or poised TSS (Figure 6D, orange). This dramatic shift in GFI1B targeting suggests that the cooperative interaction between GATA2 and GFI1B may be specific for the earlier stages of hematopoietic reprogramming and EHT that is lost in downstream hematopoietic progenitors.

## DISCUSSION

We show that ectopic expression of the TFs GGF induce a hemogenic program in human fibroblasts. Induced cells exhibit endothelial and hematopoietic gene expression and HSPC surface phenotypes and engraft NSG mice to produce multilineage progeny. Mirroring mouse hemogenic induction (Pereira et al., 2013), this transition is dynamic, with initial activation of angiogenic followed by hematopoietic gene signatures. We further show that GATA2 is the dominant TF and cooperates with GFI1B to engage open chromatin regions during the initial phases of reprogramming.

We show that upon induction with GGF, we readily detect many endothelial genes, including *PPARG*, *VWF*, and *FOXC2*, which have defined angiogenic functions. The induction results in the generation of a large population of CD49f+ACE+ cells, while only a more restricted population activates CD34. This is consistent with the observation that ACE+CD34− cells emerge early during human hematopoietic development and localize beneath the dorsal aorta (Sinka et al., 2012). Our data support the hypothesis that these precursor cells later give rise to ACE+CD34+ cells contained in aortic clusters. Indeed, *ITGA6* and *CD34* are GATA2 direct targets during the initial stages of hemogenic reprogramming, providing a direct mechanistic link between human hemogenic precursor phenotype and GATA2.

To faithfully recapitulate HSC specification major interest lies in defining direct human HSC precursors during ontogeny. Our data provide useful markers: CD49f is present on human LT-HSCs (Notta et al., 2011), and we show co-expression with ACE. The *VNN2* gene that encodes GPI-80 and marks fetal liver HSCs was not detected during reprogramming, nor was it a target for GATA2 or GFI1B. This is consistent with the lack of expression of GPI-80 in embryonic stem cell (ESC)-derived hemogenic endothelium (Prashad et al., 2015). Whether this gene is activated at later stages of HSC maturation remains to be investigated. It will be interesting in future studies to determine whether ACE+ cells present in the AGM (and perhaps in the first trimester human placenta) co-express CD49f during human embryonic development before they acquire CD34 expression. In addition, our datasets provide other markers that are informative and complement this phenotype such as *F11R*, shown to be present in HSC precursors in zebrafish (Kobayashi et al., 2014). We also found *ANGPTL4* and *JAG1* enriched in CD49f+ cells and then downregulated in CD34+ cells. These genes may be useful to segregate precursors from emergent human HSCs *in vivo*.

In addition to providing information for the identification of direct precursors of HSCs during development, our direct reprogramming strategy has identified pathways essential for EHT and HSC specification. We have shown that, in both mouse and human, GGF represent a conserved minimal TF network for hemogenic induction. In the mouse, we identified interferon signaling and inflammation as enriched pathways in hemogenic precursors (Pereira et al., 2013). Recently, multiple studies explored inflammation during EHT and HSC formation in mouse and zebrafish models (Espin-Palazon et al., 2018). Enrichment of this pathway during human hemogenic induction supports its importance for human EHT. Another example is the role of FOS and AP-1 in HSC specification. This was not revealed by classical genetic ablation, possibly because of compensatory effects. AP-1 is essential for human and mouse hemogenic induction (Lis et al., 2017; Pereira et al., 2013; Sandler et al., 2014) and has recently been implicated during EHT from mouse ESCs (Obier et al., 2016). Our studies corroborate that direct cellular reprogramming informs the process of HSC specification and offers tractable means to systematically interrogate pathways of human HSC formation. It will be interesting to address the role of other enriched pathways such as cytoskeletal regulation by Rho GTPase and the histamine H1 receptor pathway. Genes that are co-targeted during hematopoietic reprogramming by GATA2 and GFI1B are additional candidates for further study (e.g., *ITGA2*, *PLCL1*, *ADRA1B*, *RHOJ*, *SEMA3A*, and *PLCB1*; see Table S3 for comprehensive lists).

In the mouse, lineage divergence from endothelium may occur before extensive formation of intra-aortic clusters (Rybtsov et al., 2014; Swiers et al., 2013). The enrichment of angiogenesis and cell motility during human hemogenic reprogramming suggests that human hemogenic precursors may not represent a cohort of mature endothelium but more likely a different lineage with endothelial features that is committed to the hematopoietic route. This is also supported by studies of human ESC-derived hematopoiesis (Ditadi et al., 2015). Glycolysis-associated genes were rapidly activated 2 days after induction, and motif enrichment analysis identified HIF1 as a regulator of human hematopoietic reprogramming. This suggests that a metabolic shift toward glycolysis underlies hemogenic reprogramming and human stem cell formation that may be mediated by HIF and AP-1, as recently suggested in zebrafish (Harris et al., 2013; Zhang et al., 2018).

In contrast to induced PSC (iPSC) reprogramming, the mechanisms underlying direct hematopoietic reprogramming remain poorly understood. We have explored the regulatory mechanisms underlying hemogenic induction with ChIP-seq at the initial stages of reprogramming. Two different models of action have been proposed for how TFs access chromatin to initiate reprogramming: (1) pioneer TFs, as a unique class of transcriptional regulators with the capacity to bind nucleosomal DNA (closed chromatin) and activate gene regulatory networks in target cells (Soufi et al., 2012), and (2) cooperative interaction among multiple TFs (Chronis et al., 2017). In this recent study, iPSC reprogramming factors bind cooperatively to enhancer regions to direct somatic inactivation and pluripotent gene expression initiation (Chronis et al., 2017). Our data support a model (Figure 6E) whereby GGF cooperate to silence fibroblast-specific program and gradually impose the hemogenic program. GATA2 and GFI1B binding occurs at open chromatin, promoters, and enhancer regions supporting the cooperative binding model. However, GATA2 showed independent targeting capacity and was crucial for the recruitment of GFI1B to target sites. Our finding mirrors the role of GATA2 during hematopoietic progenitor differentiation by recruiting GATA1 to erythroid genes (May et al., 2013), suggesting that GATA2 nucleates the binding of the complex to initiate hematopoietic reprogramming at open chromatin regions. It is then likely that the reprogramming cascade initiated by the TFs will then rely on a stepwise process of chromatin remodeling to shut down fibroblast enhancers and open endothelial and hematopoietic enhancers, as recently demonstrated during iPSC reprogramming (Knaupp et al., 2017; Li et al., 2017). During mouse ESC differentiation, it was shown that AP-1 motifs were enriched in open chromatin regions and co-localized with TF-binding sites that were specific to hemogenic endothelial cells (Goode et al., 2016). In human endothelial cells, AP-1 cooperates with GATA2 to induce key endothelial and inflammatory genes (Kawana et al., 1995; Linnemann et al., 2011). In contrast, Gfi1b is not a part of the heptad of TFs in mouse hematopoietic progenitors (Wilson et al., 2010). Indeed, we have confirmed that in human hematopoietic progenitors, GFI1B has a very different binding pattern from GATA2 and FOS. We propose that GATA2 and GFI1B interaction is specific during hemogenic reprogramming and HSC specification. Recent ChIP-seq data from mouse ESC-derived hemogenic endothelial cells supports a similarity between GATA2 and GFI1B target sites (Goode et al., 2016). Taken together, these data highlight the cooperative action between GGF during human hematopoietic reprogramming, initially binding at fibroblast-specific genes and then activating endothelial and hematopoietic gene signatures.

Several recent publications have induced mouse and human cells into HPCs (Batta et al., 2014; Lis et al., 2017; Pereira et al., 2013; Riddell et al., 2014; Sandler et al., 2014). Two recent studies showed the generation of long-term reconstituting HSCs derived from mouse endothelial cells or human PSCs (Lis et al., 2017; Sugimura et al., 2017). In both cases, reprogrammed cells were taken into either the bone marrow niche or co-cultured on endothelial cells to promote maturation. It will be interesting to address the impact of a supportive niche on our reprogrammed cells to mature these populations. In addition, combining GATA2-, FOS-, and GFI1B-specifying factors with factors that may promote HSC maturation should be investigated. A recent study demonstrated the generation of serially engraftable murine HSCs by GGF overexpression within teratoma (Tsukada et al., 2017). This suggests that GGF are the instructive factors of HSC identity not only from fibroblasts but also from other cell types and highlights the importance of the *in vivo* environment as well as the need for suitable mouse models that support the maintenance of human HSCs (Cosgun et al., 2014). It will be of critical importance to develop more defined culture methods for the controlled maturation of *in vitro*-programmed as well as human embryo-derived nascent HSCs into definitive, fully functional HSCs.

Collectively, our results show that GGF are sufficient for the generation of hemogenic cells from human fibroblasts. Our results suggest that HSC specification is controlled by the cooperative action of TFs and underscore the importance of GATA2-GFI1B interaction and initial engagement at open chromatin regions. In summary, we demonstrate that direct cellular reprogramming provides insights into the molecular mechanisms of human HSC specification. These studies provide a plat-form for the development of patient-specific HSPCs from easily accessible HDFs.

## STAR★METHODS

### CONTACT FOR REAGENT AND RESOURCE SHARING

Further information and requests for resources and reagents should be directed to and will be fulfilled by the Lead Contact, Carlos-Filipe Pereira (filipe.pereira@med.lu.se ).

### EXPERIMENTAL MODEL AND SUBJECT DETAILS

#### Cell lines

Human adult dermal fibroblasts (HDF, ScienCell), neonatal foreskin fibroblasts (BJ) and HEK293T cells (ATCC) were grown in fibroblast media (FM media; Dulbecco’s Modified Eagle Medium (Invitrogen) containing 10% fetal bovine serum (Benchmark), 1mM L-Glutamine and penicillin/streptomycin (10 μgml^−1^, Invitrogen) in 5% (v/v) CO_2_ at 37°C. Cells were grown for 2–3 days until confluence, dissociated with TrypLE Express and frozen in Fetal Bovine Serum (FBS) 10% dimethyl sulfoxide (DMSO, Sigma). HDF and BJ were used at between passages 1–4 and 10–15, respectively. All cells were maintained at 37°C and 5% (v/v) CO_2_. All tissue culture reagents were from Thermo Fisher Scientific unless stated otherwise.

#### Mice and *in vivo* animal studies

NSG (NOD.Cg-*Prkdc*^*Scid*^*Il2rg*^*tm1Wjl*^/Sz) mice (*Mus musculus*) were ordered from Jackson laboratories and housed in the centralized animal care facility of the Center for Comparative Medicine and Surgery (Mount Sinai, New York). Animal experiments and procedures were approved by the Institutional Animal Care and Use Committee and conducted in accordance with the Animal Welfare Act. Mice were housed in groups of 3–5 at 22 C°–24 C° using a 12-h light/12-h dark cycle. Animals had *ad libitum* access to water and the regular chow diet at all times. Water was replaced by Dox supplemented water (1mg/ml) after transplantation for 2 weeks. For all experiments only female mice were used, and transplantation was performed in mice with the age of 4 weeks.

### METHOD DETAILS

#### Molecular Cloning and Lentivirus Production

Coding regions of human GFI1B, FOS and ETV6 were individually cloned into the pFUW-tetO lentiviral vector where expression is under the control of the tetracycline operator and a minimal CMV promoter. Lentiviral vectors containing the reverse tetracycline transactivator M2rtTA under the control of a constitutively active human ubiquitin C promoter (FUW-M2rtTA) and pFUW-tetO-GATA2 have been previously described (Pereira et al., 2013). The coding region of human GFI1B was inserted into the lentiviral plasmid pLV-TRE-HA. The coding region of GATA2 was inserted into pJW321–3xFLAG and sub-cloned into the pFUW-tetO vector. The primers used for cloning are listed in the Key Resource Table. 293T cells were transfected with a mixture of viral plasmid and packaging constructs expressing the viral packaging functions and the VSV-G protein. Viral supernatants were harvested after 36, 48 and 72 hours, filtered (0.45 μm) and concentrated 40-fold with Amicon ultra centrifugal filters (Millipore).

#### Viral Transduction and Cell Culture

Fibroblasts were seeded at a density of 25,000 cells per well on 0.1% gelatin coated 6-well plates and incubated overnight with pools of pFUW lentiviruses in FM media supplemented with 8 μgml^−1^ polybrene. Equal MOIs of individual viral particles were applied. Transductions with mOrange in pFUW-tetO resulted in > 95% efficiency. After 16–20 hours media was replaced with fresh FM media supplemented with Doxycycline (1 μgml^−1^). At day 4 post-transduction cells were dissociated with TrypLE Express and 10,000 cells per well were plated on 0.1% gelatin coated 6-well plates. Reprogramming cultures were maintained in Myelocult Media (H5100; Stem Cell Technologies) supplemented with Hydrocortisone (10^−6^ M; Stem Cell Technologies). Media was changed every 4 days for the duration of the cultures.

#### Immunofluorescence

Live immunofluorescence was performed with Phycoerythrin (PE)-conjugated sterile rat monoclonal antibodies against CD49f and CD34 (Key Resource Table) at a 1:20 dilution. Emergent colonies were washed once with PBS 5% FBS and incubated with conju-gated antibodies for 30 min at room temperature in the presence of mouse serum. Cultures were then washed twice with PBS 5% FBS to remove unbound antibody. For detection of tagged TFs, fibroblasts were fixed with 2% PFA for 20 min, permeabilized with 0.4% Triton X-100, blocked for 30 min and incubated with anti-FLAG, anti-HA, anti-FOS antibodies at 1:200 dilution for 2 hours. Cells were washed, incubated with secondary antibodies conjugated with Alexa Flour 488 (Invitrogen, A12379) and nuclear counterstained with 4,6-diamidino-2-phenylindole (DAPI, 1 μgml^−1^, Sigma). Cells were visualized on a Leica DMI4000 microscope and processed with Leica software and Adobe Photoshop.

#### Flow Cytometry Analysis and Fluorescence-Activated Cell Sorting

Cell cultures were dissociated with TrypLE Express or Accutase Cell detachment solution (Innovative Cell Technologies, Inc) and stained with fluorochrome-coupled antibodies (Key Resource Table). Cell populations were isolated on an InFlux cell sorter (BD Biosciences) and immediately lysed in Trizol (Ambion) for RNA extraction, cultured on 0.1% gelatin coated 6-well plates in Myelocult media or transplanted. Flow cytometric analysis was performed on a 5-laser LSRII with Diva software (BD Biosciences) and further analyzed using FlowJo software. DAPI (1 μgml^−1^) was added before analysis to exclude dead cells.

#### Long-term Repopulation Assays

NSG (NOD.Cg-*Prkdc*^*Scid*^*Il2rg*^*tm1Wjl*^/Sz, Jackson laboratories) mice were used as recipients for induced cells. For isolating a population that include both CD49f+ and CD34+ cells, cultures were dissociated with Accutase 25 days after transduction and stained with PE-conjugated anti-CD49f and PE-conjugated anti-CD34. The PE-positive cell population was isolated by FACS sorting. Animals were transplanted with cells intravenously by retro-orbital injection and administered with Dox (1mg/ml) in drinking water for 2 weeks. Up to 6 hours before transplantation with human cells 4-week old female NSG mice received a sublethal total body irradiation dose of 200 cGy. The numbers of PE+ cells transplanted were in the range of 100,000 cells per animal. Starting 3–4 weeks after transplantation, NSG mice were bled from the orbital venous plexus and human contribution was assessed by flow cytometry with mouse and human anti-CD45 antibodies and by PCR with human specific primers to the Cr17 alpha-satellite sequence. Hematopoietic lineage contribution was assessed by flow cytometry with anti-CD3, anti-CD19, anti-CD14 and anti-CD11c human antibodies.

#### Genomic PCR

Genomic DNA was isolated using Easy DNA extraction kit (Invitrogen). Presence of human sequences was checked by PCR using Phusion Flash (Thermo Scientific) high-fidelity PCR Master Mix (30 cycles of 98°C for 1 s; 60°C for 5 s and 72°C for 15 s) with primers for the chromosome 17 alpha satellite (Key Resource Table).

#### GPSforGenes

Gene expression data was downloaded from BioGPS database (GeneAtlas U133A), transformed to log-space and normalized to bring the expression values to 0–1 range for each gene across different samples. The resulting data was then searched for samples with the highest averaged expression for (GATA2 + FOS + GFI1B) and (GATA2 + FOS + GFI1B + ETV6).

#### mRNA-seq Library Preparation and Sequencing

FACS isolated cells were lysed in Trizol (Ambion). RNA integrity was evaluated using a Eukaryotic RNA 6000 Nano chip on an Agilent 2100 Bioanalyzer (Agilent Technologies). Up to 1 μg of total RNA from each sample was used for library preparation with the TruSeq RNA Sample Preparation Kit (Illumina). A common adaptor was used for all samples and barcode sequences present in the reverse primer were introduced by 12–20 cycles of amplification as described (Pereira et al., 2013). Each library was assessed for quality and size distribution using an Agilent High Sensitivity Assay bioanalyzer chip and quantified by real-time PCR. Equimolar amounts of each barcoded library were mixed and single-end sequenced on an Illumina HiSeq Sequencing System.

#### Single cell mRNA-seq Library Preparation

HDF, HDF+GGF day 2, day 15 CD49f+, day 25 CD34+CD49f+ populations were FACS sorted and collected. Umbilical cord blood was obtained from the New York Blood Center and Lin-CD34+ cells were isolated using Diamond CD34 Isolation Kit (Miltenyi Biotec) according to manufacturer’s instructions. After isolation, Lin-CD34+ cells were stored in liquid nitrogen until use. cDNA synthesis was performed following the manufacturers instruction using the C1 Single-Cell Auto Prep System (Fluidigm) (Pereira et al., 2016). The cDNA reaction products were quantified using the Quant-iT PicoGreen double-stranded DNA Assay Kit (Thermo Fisher Scientific) and then diluted to a final concentration of 0.15–0.30 μgml^−1^ using C1 Harvest Reagent. Sequencing libraries were prepared using the Nextera XT DNA Library Prep Kit (Illumina). 96 single-cell libraries were mixed together. The concentration of the mixed libraries was determined using Agilent Bioanalyzer. The libraries were sequenced yielding ~0.18–5.7 Million 75-nt reads on a HiSeq 2000 platform at Girihlet, Inc.

#### Coimmunoprecipitation (Co-IP)

Nuclear extracts were prepared from HDFs with ectopic expression of 3xFLAG-tagged GATA2, HA-tagged GFI1B and FOS and incubated with 5 μg of each antibody (Key Resource Table) The immune complexes were then washed four times with the lysis buffer by centrifugation. IP/co-IP were performed using 5% of input samples. For the control IP, we used 5 μg of rabbit IgG (Key Resource Table). Samples were heated in SDS sample buffer and processed by western blotting.

#### Western Blot Analysis

Cells were lysed in RIPA-B buffer (20 mM Na2HPO4 [pH 7.4], 150 mM NaCl, 1% Triton X-100) in the presence of protease inhibitors (3 μg/ml aprotinin, 750 μg/ml benzamidine, 1 mM phenylmethylsulfonyl fluoride, 5 mM NaF and 2 mM sodium orthovanadate) and incubated on ice for 30 min with occasional vortexing. Samples were centrifuged to remove cell debris and heated in SDS sample buffer. For immunoblotting, membranes were blocked with TBST buffer (10 mM Tris-HCl (pH 7.9), 150 mM NaCl, and 0.05% Tween 20) containing 3% milk, incubated with primary antibodies, washed three times with TBST, incubated with HRP-conjugated secondary antibodies, washed three times with TBST and subsequently detected by ECL or Femto (Thermo Scientific).

#### Chromatin Immunoprecipitation (ChIP)-seq

ChIP assays were performed in HDFs transduced with a pool of 3xFLAG-tagged-GATA2, HA-tagged-GFI1B and FOS and the transgenes were induced with Doxycycline. After 48hr, 20–50×10^^^6 cells were used for each experiment and crosslinking conditions were optimized for each factor. For GATA2 and GFI1B ChIP cells were fixed with 11% formaldehyde (Sigma) at room temperature on a rotating platform for 10 min. Formaldehyde was quenched by adding of 125 mM of glycine on a rotating platform for 5 min at room temperature and cross-linked cells were washed twice in ice-cold PBS. Chromatin shearing was done using the E210 Covaris to a 150–350bp range, insoluble debris was centrifuged, then sheared chromatin fragments were incubated overnight at 4°C with antibodies coupled to 50 μl Protein G dynabeads (Invitrogen). For FOS ChIP 3 μg of antibody was used per 5–10×10^^^6 cells and for FLAG and HA 10μg of antibody per 20–50×10^^^6 cells. Beads were washed five times with RIPA buffer and once with TE containing 50 mM NaCl, and complexes eluted from beads in elution buffer by heating at 65°C and shaking in a Thermomixer. Reverse cross-linking was performed overnight at 65°C. Whole cell extract DNA was treated for cross-link reversal. Immunoprecipitated and whole cell extract DNA were treated with RNaseA, proteinase K and purified using Phenol:Chloroform:Isoamyl Alcohol extraction followed by ethanol precipitation. For FOS ChIP, 5–10×10^^^6 cells were double crosslinked. First, cells were crosslinked in PBS supplemented with Di(N-succinimidyl) glutarate (DSG, ThermoFisher Scientific 20593) at a final concentration of 2 mM for 45 min at room temperature on a rotating platform. After 3 washes in PBS, formaldehyde crosslinking of proteins and DNA was done for 10 min at room temperature at a concentration of 11% formaldehyde (Sigma) in PBS. Formaldehyde was quenched by adding of 125 mM of glycine on a rotating platform for 5 min at room temperature and crosslinked cells were washed twice in ice-cold PBS. Libraries were prepared using either KAPA Hyper Prep Kit or NEBNext ChIP-seq Library Prep Master Mix Set for Illumina according to the manufacturer’s guidelines. Libraries were size-selected on a 2% agarose gel for a 200–400bp fragments and were sequenced on Illumina HiSeq 2000.

#### ChIP-seq Data Visualization

To produce the heatmaps, each feature (such as peaks of a TF, histone marks) was aligned at GATA2 or GFI1B summits and tiled the flanking up- and downstream regions within ± 4kb in 100bp bins. To control for input in our data, we computed at each bin a input-normalized value as log2(RPKM_Treat_) - log2(RPKM_Input_), where RPKM_Treat_ is RPKM of the corresponding TF or histone and RPKM_Input_ is RPKM of the corresponding whole genome ‘Input’. We plotted the density of DNase-seq signal within ± 1kb around the center of GATA2 or GFI1B summits and compared it to the resistant sites, which were resized to be in the same range as GATA2 or GFI1B summits.

### QUANTIFICATION AND STATISTICAL ANALYSIS

#### mRNA-seq Analysis

For each sample 4.5–26.5 M 100-nt reads were obtained, pre-processed with the FASTX-toolkit suite and aligned to the human genome (*Homo sapiens* hg19 assembly) using TopHat mapper. Post alignment with TopHat release 1.4.1 against the *Homo sapiens hg19* assembly using the known transcripts option. All resultant .bam files were processed using Samtools version 0.2.5 and Bed-tools version 2.16.2 and visualized on the Integrated Genome Browser version 2.1 or the UCSC Genome Browser. Transcript assembly and expression estimation was conducted with Cufflinks release 1.3.0 using a *Homo sapiens hg19* reference annotation and upper quartile normalization. Cufflinks assemblies were merged and processed through Cuffdiff for gene FPKM reporting and differential expression analysis. Each library was treated as a separate non-replicate sample. Gene transcript count data from the mRNA-seq analysis was obtained by reprocessing the data through TopHat release 2.0.0 and Cufflinks and Cuffdiff release 2.0.0. Gene set enrichment analysis (GSEA) between HDF or BJ and CD34+CD49f+ was performed using the genes.fpkm.tracking file (Table S1) output from Cufflinks release 1.3.0 run against the Molecular Signatures Database version 2.0 curated gene sets (Gene set sizes 0–5000) ranked by Ratio_of_Classes. Non-negative Matrix Factorization (NMF) of the FPKM values obtained from RNA sequencing was performed on the GenePattern Platform using the NMF consensus analysis module at *k. initial* = 2 and *k. final* = 5, here we show metagene results for *k* = 4. Visualization of FPKM expression density and inter-sample FPKM correlation was conducted in R version 2.15.0 with the CummeRbund package. Gene list enrichment analysis with gene set libraries created from level 4 of the MGI mouse phenotype ontology was performed with Enrichr.

#### Single cell mRNA-seq Analysis

For single cell mRNA-Seq analysis the raw fastq files were aligned against the Ensemble GRCh38 genome using the Gencode v25 gene annotation. Gene level read counts were then calculated using featureCounts from the Subread package. Raw counts were log transformed and quantile normalized. For hierarchical clustering Pearson correlation was used as distance metric. Hierarchical clustering was plotted using the dendextend library in R.

#### mRNA-seq Quality Control

Scater library (McCarthy et al., 2017) was used to include samples and genes that pass quality control. For single cell analysis, we first discarded genes that are expressed in less than 1% of the cells. Then we applied Scater library function ‘isOutlier’ with 3 median absolute deviations in order to define the thresholds for following parameters: total number of counts, number of genes detected and number of counts belonging to mitochondrial genes. Values beyond this threshold are considered outliers and were not included in the analysis. As a result, we defined the following criteria: 1) total number of counts detected per sample ≥ 891,010; 2) number of genes detected in each single cell ≥ 2,619; 3) number of counts belonging to mitochondrial genes ≤ 500,224. From single cell mRNA-seq 39 cells did not pass the quality control filters and were not included in the analysis. As for genes, from 56,269 we used 24,536 for analysis. For population mRNA-seq from the initial 24 samples from BJ and HDF-derived cells 1 sample did not pass the quality control and was discarded (BJ Day 15 CD49f+).

#### ChIP-seq analysis

ChIP-seq analysis was performed on the raw FASTQ files. FASTQ files were mapped to the human *hg19* genome using Bowtie 2 program allowing for 2 base pair mismatches. Mapped output files were processed through MACS1.4 analysis software to determine peaks. Homer software package was used for peak annotation and further analysis was performed using the Galaxy server and R statistical software.

#### Chromatin State Fold-Enrichment

Enrichment scores for genomic features, such as GATA2 and GFI1B Chip-seq peaks and histone marks were calculated using ChromHMM Overlap Enrichment (Chronis et al., 2017), based on public segmentation. ChromHMM segmentation, that contains 18 different chromatin states, was downloaded from Roadmap website and used for analysis. Enrichment scores were calculated as the ratio between the observed and the expected overlap for each feature and chromatin state based on their sizes and the size of the human genome.

#### Gene List Enrichment Analysis with Single Cell Data

Differential gene expression was calculated for all pairwise sample groups (HDF, DAY2, CD34+ CD49f+, CD49f+, UCB CD34+) using variance-stabilizing transformation combined with limma. To calculate gene set enrichment the top 500 and bottom 500 genes for each differential expression gene signature were uploaded to Enrichr. The significance of gene set overlap was measured using the Fisher Exact test.

#### Motif Analyses

For *de novo* motif discovery, findMotifsGenome.pl procedure from Homer was used on GATA2 and GFI1B separately. Co-bound regions by GFI1B and GATA2 were found using bedtools. Co-bound regions were used for *de novo* motif discovery using Homer and CCAT. In order to evaluate similarity of the two sets based on the intersections Jaccard statistic were used.

#### Integration of Independently Obtained Gene Expression and Genome Location Datasets

Data from microarray and RNA-seq experiments were adjusted using quantile normalization; genes insignificantly changing their expression across samples were removed using ANOVA with the consequent adjustment for multiple testing (Benjamini-Hochberg correction, p < 0.05). Data for the remaining genes were converted to Z-scores. Hierarchical clustering and Principal Component Analysis (PCA) of the integrated dataset was performed using Cluster 3.0 and visualized with JAVA treeview. For differential gene expression analysis, DESeq2 was used in HDF, day 15 CD49f+, day 25 CD49f+ and day 25 CD34+CD49f+ and genes were selected using the following criteria: 1) DESeq2 differential calls with an adjusted p value < 0.05 (FDR used as adjusted p value) between either of four groups of sample; 2) Absolute changes in expression between minimal and maximal expression > 1.5 fold. Intersection between the genes identified by Chip-Seq for GATA2 and GFI1B (binding peaks within ± 5kb around the transcriptional start) and mRNA-seq identified 1,425 genes, which were clustered and visualized. Those 1,425 genes we divided into 3 groups: 1) Genes bound only by GATA2; 2) Genes bound only by GFI1B; 3) Genes co-bound by GATA2 and GFI1B.

### DATA AND SOFTWARE AVAILABILITY

The accession number for population, single cell mRNA-seq, and ChIP-seq data reported in this paper is Gene Expression Omnibus (GEO): GSE51025. The public datasets used in this study can be found in Table S4.

## Supplementary Material

1

2

3

4

## Figures and Tables

**Table:** KEY RESOURCES TABLE

REAGENT or RESOURCE	SOURCE	IDENTIFIER
Antibodies		
Mouse Anti-Human CD34 clone 581	BD Biosciences	Cat#560710; RRID: AB_2687922
Mouse Anti-Human CD34 clone 8G12	BD Biosciences	Cat#348057; RRID: AB_2687922
Rat Anti-Human CD49f clone GoH3	BD Biosciences	Cat#555736; RRID: AB_396079
Rat anti-mouse CD45 clone 30-F11	Bio Legend	Cat#103126; RRID: AB_493536
Mouse Anti-Human CD45 clone HI30	BD Biosciences	Cat#560777; RRID: AB_1937324
Mouse Anti-Human CD143 (ACE) clone BB9	BD Biosciences	Cat#557813; RRID: AB_396883
Mouse Anti-Human CD90 clone 5E10	eBioscience	Cat#17–0909-42; RRID: AB_953611
Mouse Anti-Human CD38 clone HIT2	BD Biosciences	Cat#560676; RRID: AB_1727472
Biotin Anti-Human CD133 clone AC133	Miltenyi Biotec	Cat#130–090-664; RRID: AB_244341
Mouse Anti-Human CD3 clone UCHT1	eBioscience	Cat#47–0038-42; RRID: AB_906221
Mouse Anti-Human CD19 clone HIB19	Biologend	Cat#302254; RRID: AB_2564142
Mouse Anti-Human CD11c clone 3.9	Biologend	Cat#301618; RRID: AB_439791
Mouse Anti-Human CD14 clone HDC14	Biologend	Cat#325618; RRID: AB_830691
Diamond human CD34 isolation kit	Miltenyi Biotec	Cat#130–094-531; RRID: AB_2721154
Mouse Monoclonal Anti-FLAG clone M2	Sigma Aldrich	Cat#F1804; RRID: AB_259529
Rabbit polyclonal Anti-HA clone 4C12	Abcam	Cat#ab9110; RRID: AB_10637297
Rabbit polyclonal Anti-FOS clone 4	Santa Cruz	Cat#sc-52; RRID: AB_2106783
Normal rabbit IgG	Santa Cruz	Cat#sc-2025; RRID: AB_737196
Mouse monoclonal β-actin clone AC-74	Sigma Aldrich	Cat#A2228; RRID: AB_476697

Bacterial and Virus Strains		

Competent *E. coli* DH5α	NEB	Cat#C2987I

Biological Samples		

Cord Blood	New York Blood Center	N/A

Chemicals, Peptides, and Recombinant Proteins		

Polybrene	Sigma	Cat#H9268
Doxycycline hyclate	Sigma	Cat#D9891
Dulbecco’s Modified Eagle Medium (DMEM)	GIBCO	Cat#11965–092
L-Glutamine	GIBCO	Cat#25030–081
Penicillin-Streptomycin Solution	GIBCO	Cat#15140–122
FBS	BenchMark	Cat#100–106
TryPLE Express	GIBCO	Cat#12605–010
Accutase Cell detachment solution	Innovative Cell Technologies	Cat#AT104
Dulbecco’s Phosphate Buffer Solution (DPBS)	GIBCO	Cat#14190–144
Myelocult Media	Stem Cell Technologies	Cat#5150
Hydrocortisone	Stem Cell Technologies	Cat#07904
Trizol Reagent	Ambion RNA	Cat#15596026
ECL	Thermo Scientific	Cat#32209
Femto	Thermo Scientific	Cat#34095
Protein G Agarose beads	Roche	Cat# 11719416001
Protein G Dynabeads	Life Technologies	Cat#10004D
Proteinase K	Invitrogen	Cat#100005393
RNase A	5Prime	Cat#2900403
Phenol/Chloroform/Isoamyl Alcohol	Fisher	Cat#BP1752
Sodium Acetate	Sigma	Cat#S7899
GlycoBlue	Life Technologies	Cat#AM9515
Glycine	Fisher	Cat#G48–212
Formaldehyde solution	Sigma	Cat#F8775
Gelatin from Porcine Type A	Sigma	Cat#G1890–100
Molecular grade water	Corning	Cat#46–000-1
BSA	Fisher	Cat#BP1600
BES buffered saline solution	Sigma	Cat#14280–100
Protease Inhibitor cocktail tablets	Roche	Cat#1187358001

Critical Commercial Assays		

Nextera XT library preparation kit	Illumina	Cat#FC-121–1031
TruSeq RNA Sample Prep kit	Illumina	Cat#RS-122–2001
KAPA Hyper Prep Kit	KAPA Biosystems	Cat#KK8502
NEBNext ChIP-seq Library Prep Master Mix Set	New England Biolabs	Cat#E6240L
Agilent High Sensitivity DNA kit	Agilent Technologies	Cat#5067–4626
Agilent RNA 6000 Nano kit	Agilent Technologies	Cat#5067–1511
Diamond CD34 Isolation kit	Miltenyi Biotec	Cat#130–094-531
C1 Single-Cell Reagent kit	Fluidigm	Cat#100–6201
SMARTer Ultra Low RNA kit for the Fluidigm C1 System	Clontech	Cat#634833
Quant-iT PicoGreen double-stranded DNA Assay kit	Thermo Fisher Scientific	Cat#P7589
AMPure XP beads	Beckman Coulter	Cat#A63881

Deposited Data		

Bulk RNA-seq, Single Cell RNA-seq, ChIP-seq data	This paper	Gene Expression Omnibus (GEO): GSE51025

Experimental Models: Cell Lines		

Human adult dermal fibroblasts (HDF)	ScienCell	Cat#3220
Neonatal foreskin fibroblasts (BJ)	ATCC	Cat#CRL-2522
HEK293T	ATCC	Cat#CRL-3216

Experimental Models: Organisms/Strains		

NSG (NOD.Cg-*Prkdc*^*Scid*^*Il2rg*^*tm1Wjl*^/Sz)	Jackson laboratories	Cat#005557; RRID: IMSR_JAX:005557

Oligonucleotides		

GATA2 NheI F (pJW321–3xFLAG) – AATATCGCTAGCatg gag gtg gcg ccc gag cag ccg	This paper	N/A
GATA2 PacI R (pJW321–3xFLAG) – GGTATCTTAATTAA tcaCTAGCCCATGGCGGTCAC	This paper	N/A
GFI1B HpaI F (pLV-HA) – AATATCGTTAACATGCCACG CTCCTTCCTG	This paper	N/A
GFI1B ClaI R (pLV-HA) – GGTATCATCGATTCACTTGA GATTGTGCTGGCT	This paper	N/A
GFI1B EcoRI F (pFUW) – AATATCGAATTCATGCCAC GCTCCTTCCTG	This paper	N/A
GFI1B EcoRI R (pFUW) – GGTATCGAATTCTCACTTG AGATTGTGCTGGCT	This paper	N/A
FOS EcoRI F (pFUW) – AATATCGAATTCATGATGTTC TCGGGCTTCAACGCAG	This paper	N/A
FOS EcoRI R (pFUW) – GGTATCGAATTCTCACAGG GCCAGCAGCGTGGG	This paper	N/A
CR17AS 5*’* – GGGATAATTTCAGCTGACTAAACAG	This paper	N/A
CR17AS 3*’* – TTCCGTTTAGTTAGGTGCAGTTATC	This paper	N/A

Recombinant DNA		

pFUW-M2rtTA	Pereira et al., 2013	N/A
pFUW-tetO-GATA2	Pereira et al., 2013	N/A
pFUW-tetO-FOS	This paper	N/A
pFUW-tetO-GFI1B	This paper	N/A
pFUW-tetO-ETV6	This paper	N/A
pLV-tetO-HA-GFI1B	This paper	N/A
pFUW-tetO-3xFLAG-GATA2	This paper	N/A
pJW321–3xFlag-NANOG	Given by Wang lab	N/A
pLV-TRE-HA-GFP	Given by Lemischka lab	N/A

Software and Algorithms		

GPSforGenes	This paper	N/A
FASTX tool kit	N/A	http://hannonlab.cshl.edu/fastx_toolkit/
Homer	Heinz et al., 2010	http://homer.ucsd.edu/homer/
Bedtools	Quinlan and Hall, 2010	http://bedtools.readthedocs.io/en/latest/
Samtools	Li et al., 2009	http://www.htslib.org/
Cufflinks	Trapnell et al., 2012	http://cole-trapnell-lab.github.io/cufflinks/
Enrichr	Chen et al., 2013	http://amp.pharm.mssm.edu/Enrichr/
MACS1.4	Zhang et al., 2008	http://liulab.dfci.harvard.edu/MACS/00README.html
TopHat	Trapnell et al., 2009	https://ccb.jhu.edu/software/tophat/index.shtml
FASTQC	N/A	https://www.bioinformatics.babraham.ac.uk/projects/fastqc/
ChromHMM	Ernst and Kellis, 2012	http://compbio.mit.edu/ChromHMM/
Limma Voom	R project for Statistical Computing	http://bioconductor.org/packages/release/bioc/html/limma.html
Galaxy	Galaxy server	https://usegalaxy.org
UCSC Genome Browser	N/A	https://genome.ucsc.edu
Gene set enrichment analysis (GSEA)	Subramanian et al., 2005	http://software.broadinstitute.org/gsea/index.jsp
DESeq2	R project for Statistical Computing	https://bioconductor.org/packages/release/bioc/html/DESeq2.html
Ilustrator	Adobe	http://www.adobe.com/cn/products/cs6/illustrator.html
Photoshop	Adobe	http://www.adobe.com/cn/products/cs6/photoshop.html
LSRII Diva Software	BD Biosciences	http://www.bdbiosciences.com/us/home
FlowJo Software	FlowJo, LLC	https://www.flowjo.com/
GraphPad Prism 6.0	GraphPad Software	https://www.graphpad.com/scientific-software/prism/

## Highlights

- GATA2, GFI1B, and FOS induce a hemogenic program in human fibroblasts
- Induced cells display dynamic endothelial-to-hematopoietic transition programs
- GATA2 is the dominant transcription factor that initiates hemogenic reprogramming
- GATA2 and GFI1B interact and bind cooperatively at open chromatin
