# Supplementary material for: Cooperative Transcription Factor Induction Mediates Hemogenic Reprogramming
Source: Cell Rep. Author manuscript; Available in PMC 2019 Jun 16. (PMC6571141; doi:10.1016/j.celrep.2018.11.032)
Supplement: 1 [file NIHMS1529552-supplement-1.pdf]

Cell Reports, Volume *25*

Supplemental Information

Cooperative Transcription Factor Induction Mediates Hemogenic Reprogramming

Andreia M. Gomes, Ilia Kurochkin, Betty Chang, Michael Daniel, Kenneth Law, Namita Satija, Alexander Lachmann, Zichen Wang, Lino Ferreira, Avi Ma'ayan, Benjamin K. Chen, Dmitri Papatsenko, Ihor R. Lemischka, Kateri A. Moore, and Carlos-Filipe Pereira

Cell Reports, Volume *25*

Supplemental Information

Cooperative Transcription Factor Induction Mediates Hemogenic Reprogramming

Andreia M. Gomes, Ilia Kurochkin, Betty Chang, Michael Daniel, Kenneth Law, Namita Satija, Alexander Lachmann, Zichen Wang, Lino Ferreira, Avi Ma'ayan, Benjamin K. Chen, Dmitri Papatsenko, Ihor R. Lemischka, Kateri A. Moore, and Carlos-Filipe Pereira

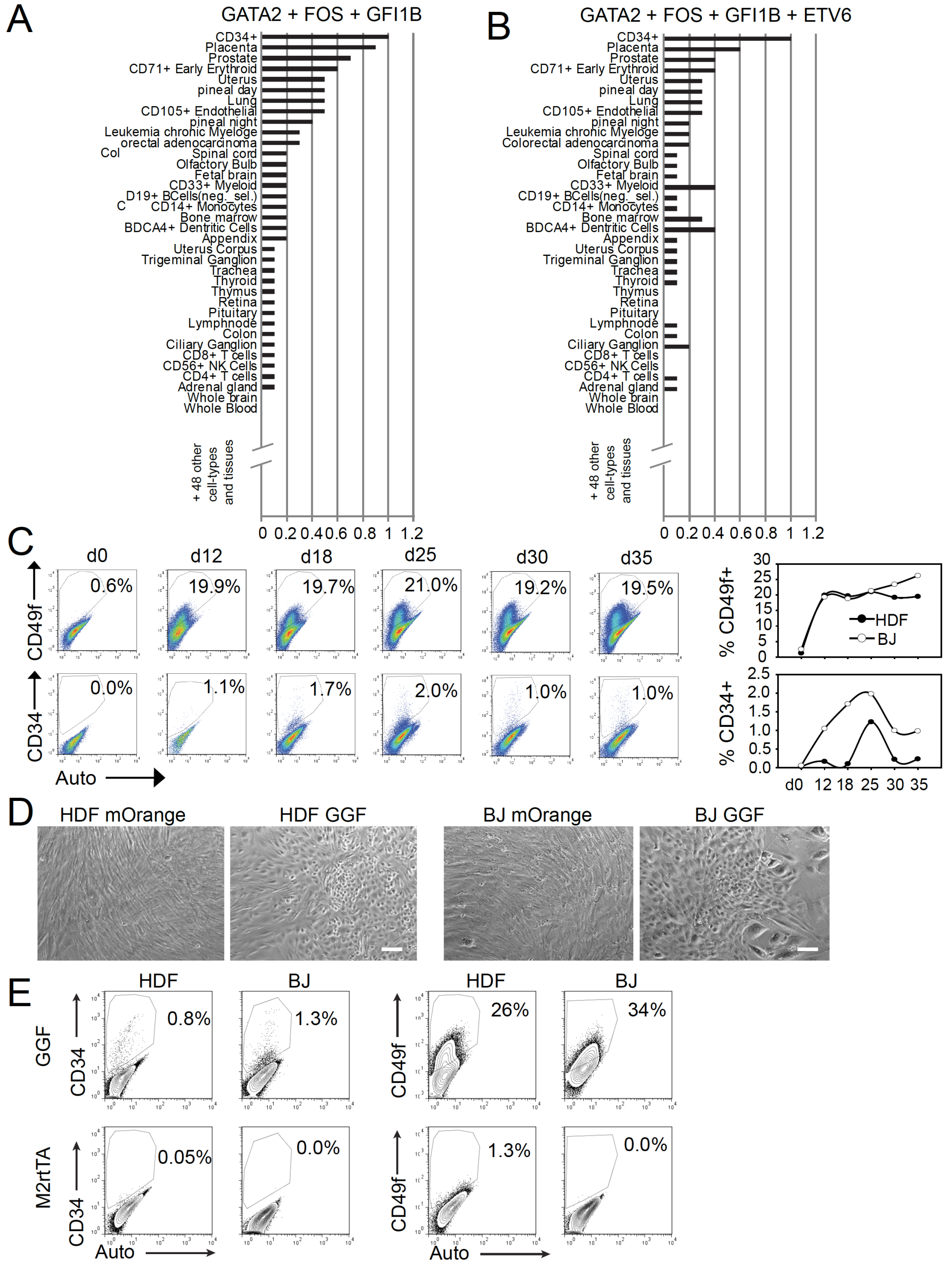

Figure S1. Analysis of transcription factor gene expression in human cells/tissues and characterization of induced cells, related to Figure 1. The combination of either (**A**) GATA2, FOS and GFI1B or (**B**) GATA2, FOS, GFI1B and ETV6 is mostly enriched in human CD34+ HSPCs among 84 tissues and cell-types. Gene expression data from different human tissues and cell-types, were obtained from the GeneAtlas U133A database. The "GPSforGenes" program was written and used to classify the tissues where combinations of TFs were most enriched (best fit = 1). (**C**) Kinetics of induction in days of CD34 and CD49f-positive populations. (**D**) Morphological analysis of HDF and BJ fibroblasts 26 days after transduction with GGF or mOrange as a control. (**E**) Flow cytometry analysis of CD34 and CD49f expression in HDF and BJ fibroblasts 26 days after transduction with GGF (upper panels) or M2rtTA as a control (lower panels).

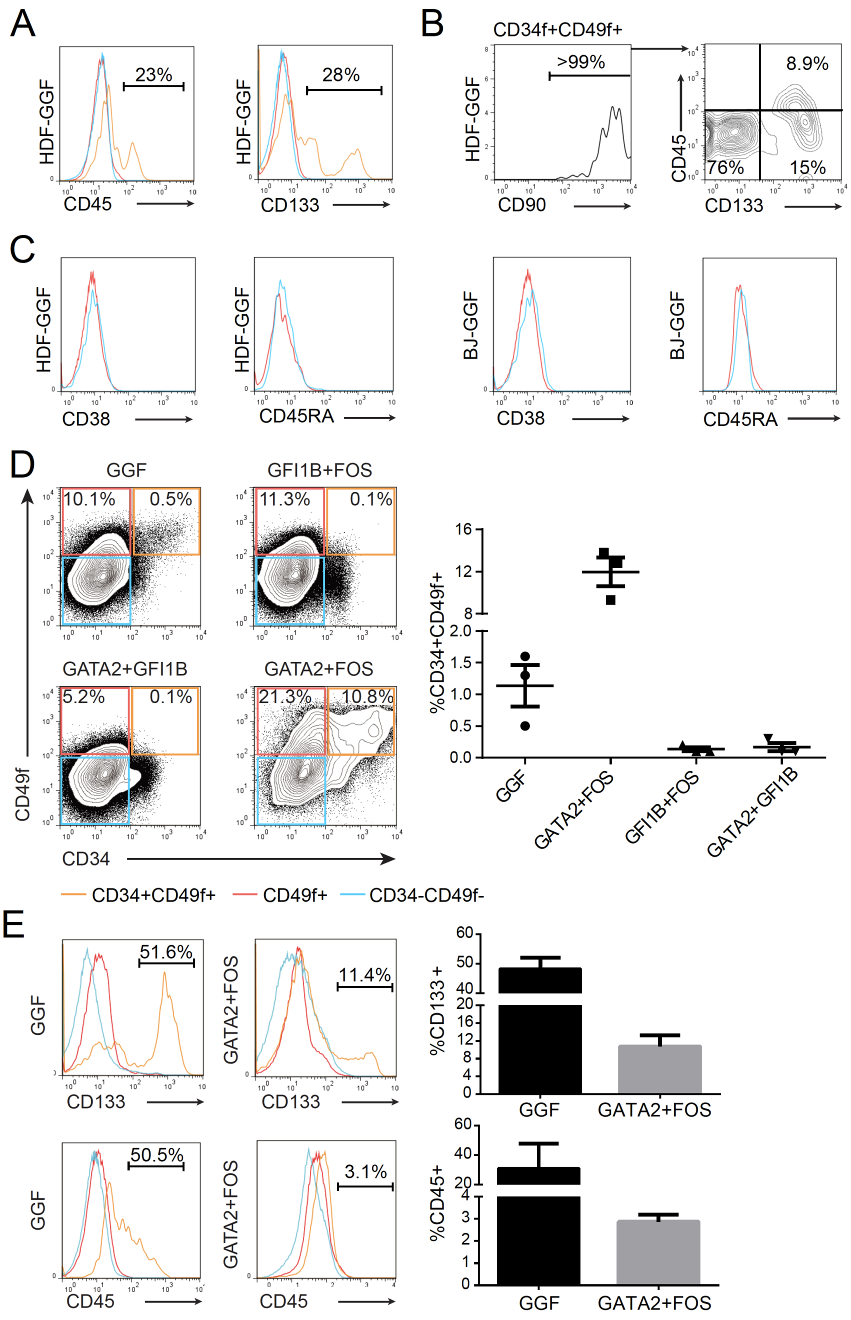

Figure S2. A human HSPC-like cell surface phenotype is induced during reprogramming, Related to Figure 1. (**A**) Expression of CD45 and CD133 within CD34+CD49f+ (orange lines), CD49f+ (red lines) and double negative population (blue lines) from two independent experiments starting with HDFs. (**B**) Expression of CD90, CD45 and CD133 within the CD34+CD49f+ population. (**C**) Lack of expression of CD38 and CD45RA in both CD34+ (red lines) and CD34- (blue lines) populations in both HDF (left panel) or BJ (right panel) derived cells from two independent experiments. (**D**) Flow cytometry analysis of CD34 and CD49f expression 27 days after Dox induction in fibroblasts transduced with GATA2, GFI1B and FOS (GGF) or with combinations of two TFs (orange box CD34+CD49f+, red box CD34-CD49f+, blue box CD34-CD49f-). Quantification of the CD34+CD49f+ population (right panel) between days 25-28 (mean ± SD, n=3). (**E**) Expression of CD45 and CD133 within the CD34+CD49f+ population (orange lines), CD49f+ single positive (red lines) or double negative populations (blue lines) in fibroblasts transduced with GGF or GATA2+FOS. Quantification of the CD133+ population (upper right panel) and CD45+ population (lower right panel) between days 25-28 (mean ± SD, n=3).

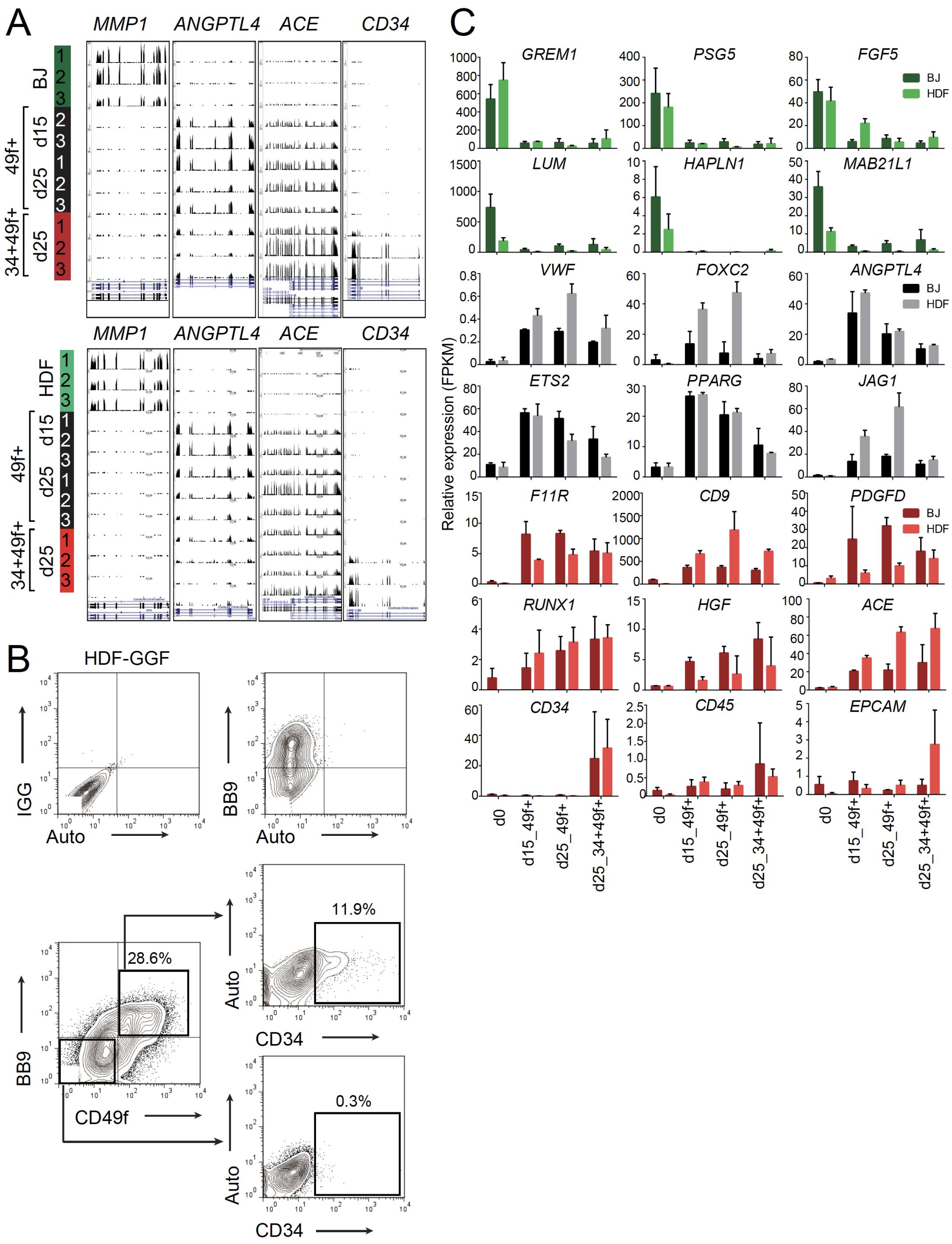

Figure S3. Key hemogenic genes are induced during reprogramming, Related to Figure 2. (**A**) Fibroblasts were transduced with GATA2, GFI1B and FOS (GGF) and global gene expression levels in non-transduced fibroblasts (BJ) and human dermal fibroblasts (HDF), day 15 CD49f+, day 25 CD49f+ and day 25 CD34+CD49f+ were profiled by RNA-seq (biological replicates: 1, 2 and 3). Reads were aligned to the human genome and those that mapped to the *MMP1*, *ANGPTL4*, *ACE* and *CD34* genes are displayed as maximum read heights. (**B**) Flow cytometry analysis with the BB9 antibody (that detects ACE cell surface expression) in HDFs 25 days after transduction with GGF. Percentages of CD49f+BB9+ and CD49f+BB9+CD34+ cells and isotype control for BB9 are shown. (**C**) The expression levels of fibroblast (green), endothelial (black and grey) and hematopoietic (red)-specific genes are shown as FPKM mean values ± SD.

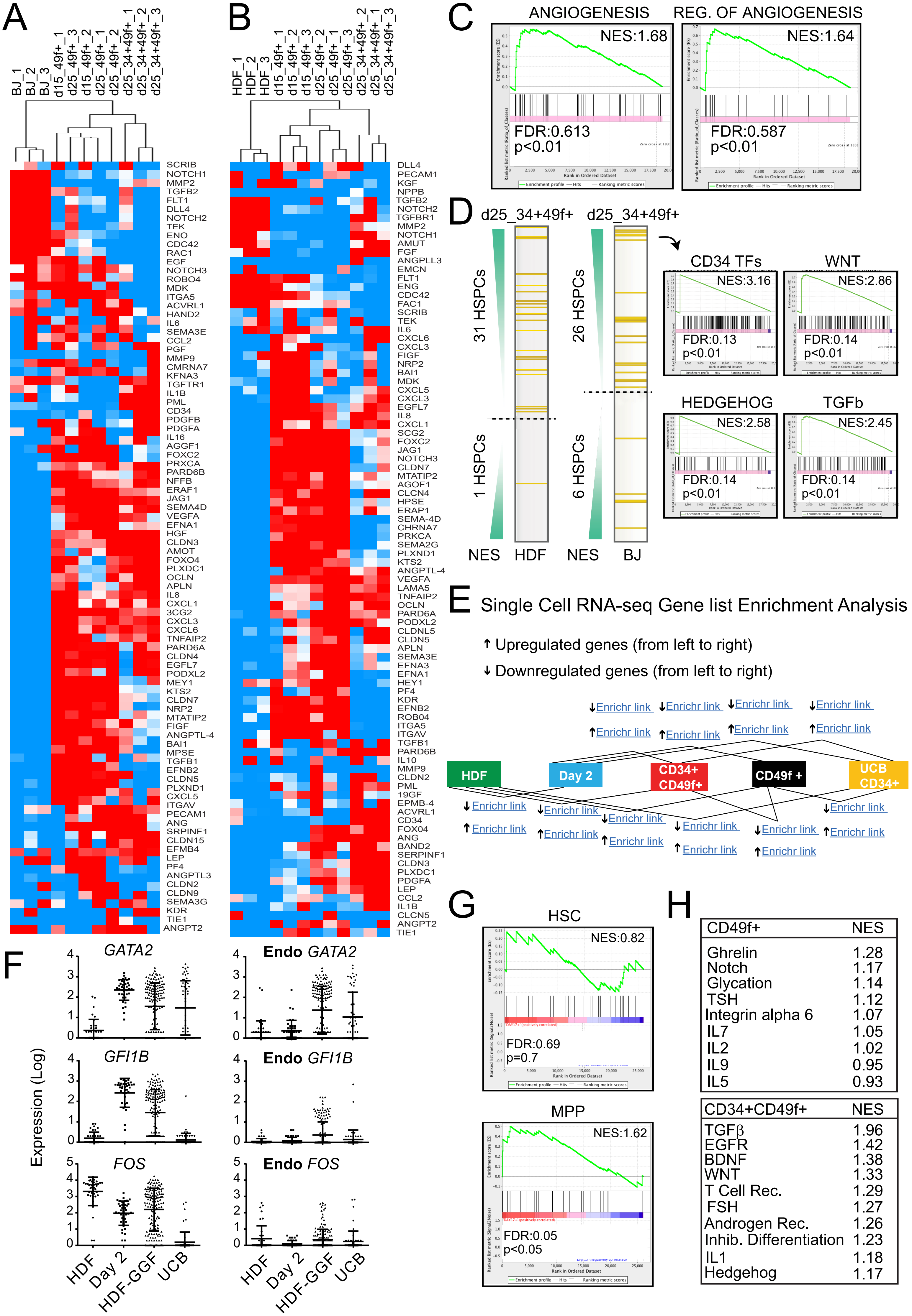
A
BJ_1
BJ_2
BJ_3
d15_49f+_1
d25_49f+_3
d15_49f+_2
d25_49f+_1
d25_49f+_2
d25_34+49f+_1
d25_34+49f+_2
d25_34+49f+_3
SCRIB NOTCH1 MMP2 TGFB2 FLT1 DLL4 NOTCH2 TEK ENO CDC42 RAC1 EGF NOTCH3 ROBO4 MDK ITGA5 ACVRL1 HAND2 IL6 SEMA3E CCL2 PGF MMP9 CMRNA7 KFNA3 TGFTR1 IL1B PML CD34 PDGFB PDGFA IL16 AGGF1 FOXC2 PRXCA PARD6B NFFB ERAF1 JAG1 SEMA4D VEGFA EFNA1 HGF CLDN3 AMOT FOXO4 PLXDC1 OCLN APLN IL8 CXCL1 3CG2 CXCL3 CXCL6 TNFAIP2 PARD6A CLDN4 EGFL7 PODXL2 MEY1 KTS2 CLDN7 NRP2 MTATIP2 FIGF ANGPTL-4 BAI1 MPSE TGFB1 EFNB2 CLDN5 PLXND1 CXCL5 ITGAV PECAM1 ANG SRPINF1 CLDN15 EFMB4 LEP PF4 ANGPTL3 CLDN2 CLDN9 SEMA3G KDR TIE1 ANGPT2
B
HDF_1
HDF_2
HDF_3
d15_49f+_1
d15_49f+_2
d15_49f+_3
d25_49f+_2
d25_49f+_1
d25_49f+_3
d25_34+49f+_2
d25_34+49f+_1
d25_34+49f+_3
DLL4 PECAM1 KGF NPPB TGFB2 NOTCH2 TGFBR1 MMP2 NOTCH1 AMUT FGF ANGPLL3 EMCN FLT1 ENG CDC42 FAC1 SCRIB TEK IL6 CXCL6 CXCL3 FIGF NRP2 BAI1 MDK CXCL5 CXCL3 EGFL7 IL8 CXCL1 SCG2 FOXC2 JAG1 NOTCH3 CLDN7 MTATIP2 AGOF1 CLCN4 HPSE ERAP1 SEMA-4D CHRNA7 PRKCA SEMA2G PLXND1 KTS2 ANGPTL-4 VEGFA LAMA5 TNFAIP2 OCLN PARD6A PODXL2 CLDNL5 CLDN5 APLN SEMA3E EFNA3 EFNA1 HEY1 PF4 KDR EFNB2 ROB04 ITGA5 ITGAV TGFB1 PARD6B IL10 MMP9 CLDN2 PML 19GF EPMB-4 ACVRL1 CD34 FOX04 ANG BAND2 SERPINF1 CLDN3 PLXDC1 PDGFA LEP CCL2 IL1B CLCN5 ANGPT2 TIE1
C
ANGIOGENESIS
NES:1.68
FDR:0.613
p<0.01
REG. OF ANGIOGENESIS
NES:1.64
FDR:0.587
p<0.01
D
d25_34+49f+
d25_34+49f+
31 HSPCs
1 HSPCs
26 HSPCs
6 HSPCs
NES
HDF
NES
BJ
CD34 TFs
NES:3.16
FDR:0.13
p<0.01
WNT
NES:2.86
FDR:0.14
p<0.01
HEDGEHOG
NES:2.58
FDR:0.14
p<0.01
TGFb
NES:2.45
FDR:0.14
p<0.01
E
Single Cell RNA-seq Gene list Enrichment Analysis
↑ Upregulated genes (from left to right)
↓ Downregulated genes (from left to right)
HDF
Day 2
CD34+ CD49f+
CD49f +
UCB CD34+
↓Enrichr link
↑Enrichr link
F
GATA2
GFI1B
FOS
Endo GATA2
Endo GFI1B
Endo FOS
Expression (Log)
HDF
Day 2
HDF-GGF
UCB
G
HSC
NES:0.82
FDR:0.69
p=0.7
MPP
NES:1.62
FDR:0.05
p<0.05
H
CD49f+ NES
Ghrelin 1.28
Notch 1.17
Glycation 1.14
TSH 1.12
Integrin alpha 6 1.07
IL7 1.05
IL2 1.02
IL9 0.95
IL5 0.93
CD34+CD49f+ NES
TGFβ 1.96
EGFR 1.42
BDNF 1.38
WNT 1.33
T Cell Rec. 1.29
FSH 1.27
Androgen Rec. 1.26
Inhib. Differentiation 1.23
IL1 1.18
Hedgehog 1.17

Figure S4. Induced hemogenic cells display an angiogenic and HSPC gene expression signature, Related to Figure 2. The expression of genes implicated in angiogenesis is shown in (**A**) BJ and (**B**) HDF derived cells. Red indicates increased expression and blue, decreased expression over the mean. Data were analyzed by Cluster 3.0 and displayed by Treeview. (**C**) Gene set enrichment analysis (GSEA) was performed for CD49f+ cells compared to BJs. Gene expression lists were analyzed for enrichment of angiogenesis and regulation of angiogenesis gene sets present in the GO biological process database. (**D**) GSEA for HDF and BJ to CD34+CD49f+ samples. Gene expression lists were analyzed for enrichment of gene sets present in the Molecular Signatures Database (MSigDB) (1888 gene sets, gene size 0-5,000). Orange lines represent HSPC datasets ordered according to normalized enrichment score (NES). The dashed line highlights the cut-off FDR = 0.25. Right panels show GSEA for CD34 enriched TFs, Wnt, Hedgehog and TGFβ signaling pathways gene sets. (**E**) Gene list enrichment analysis based on single-cell RNA-Seq data. The 500 most differentially expressed genes between HDF, day 2, day 15 CD49f+, day 25 CD49f+CD34+ and CD34+ UCB were used. Upregulated and downregulated genes and their functional enrichment from the population on the left to the population on right-hand side are displayed by *Enrichr* hyperlinks (amp.pharm.mssm.edu). (**F**) Total (left panel) and endogenous (right panel) single cell expression of *GATA2*, *GFI1B* and *FOS* is shown as Log counts presented as scatter plot for HDFs, transduced HDFs at day 2, reprogrammed CD49f+ and CD34+CD49f+ (HDF-GGF) and umbilical cord blood CD34+ cells. (**G**) GSEA was performed comparing CD34+CD49f+ cells to CD49f+. Gene expression lists were analyzed for enrichment of HSC (upper panel) and MPP (lower panel) gene sets from Notta et al 2011. (**H**) GSEA using annotated gene sets from NetPath-annotated signaling pathways highlighting top enriched pathways in CD49f+ (upper panel) and CD34+CD49f+ cells (lower panel). NES, normalized enrichment score.

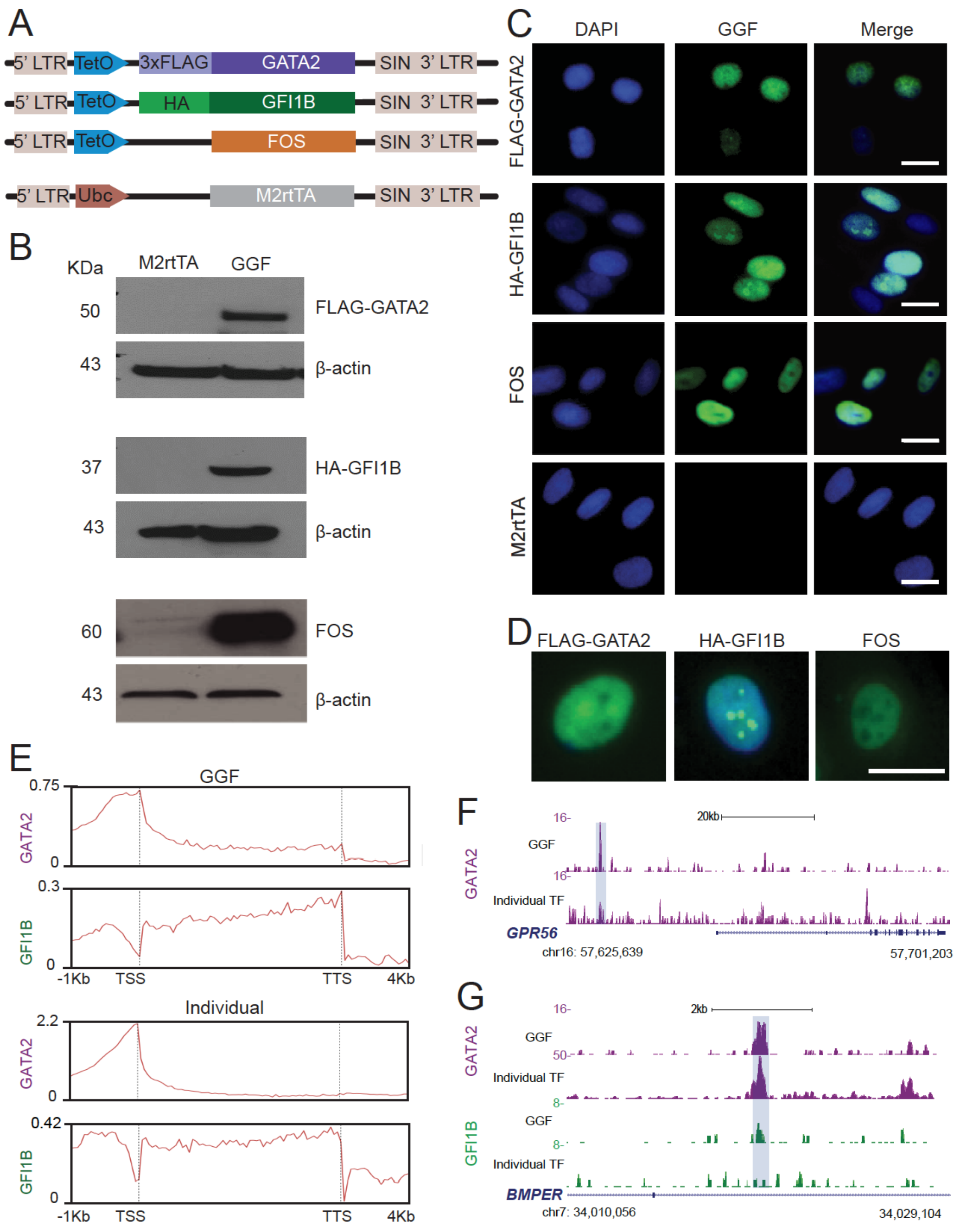

Figure S5. Strategy for detection and immunoprecipitation of GATA2, FOS and GFI1B in fibroblasts, Related to Figure 4. (**A**) Schematic representation of the lentiviral vector strategy used to transduce HDFs. (**B**) The specific detection of TFs was validated by western blotting using antibodies against FOS, FLAG or HA tags 48 hours after transducing with GATA2, GFI1B and FOS (GGF). (**C**) Transduced HDFs were assayed by immunofluorescence using antibodies against FOS, FLAG or HA tags where appropriate. Merged pictures show that FOS, FLAG-GATA2 or HA-GFI1B (Green) localize in the nucleus (Dapi, Blue) as expected. (**D**) Shows the sub-nuclear localization of the three transcription factors and highlight the binding pattern of GFI1B (middle panel). Scale bars = 20 µm. (**E**) ChIP-seq signal distribution for GATA2 and GFI1B when TFs expressed in combination (GGF, upper panels) or individually (lower panels). Plots display the average signal 1kb upstream the transcriptional start site (TSS), along gene bodies and 4kb downstream the transcription termination site (TTS). Genic regions are represented as a 3kb long metagene. (**F**) GATA2 occupancy profile at the *GPR56* locus. Genomic scale is in kilobases (kb). (**G**) GATA2 and GFI1B occupancy profile at the *BMPER* locus. Major peaks are highlighted.

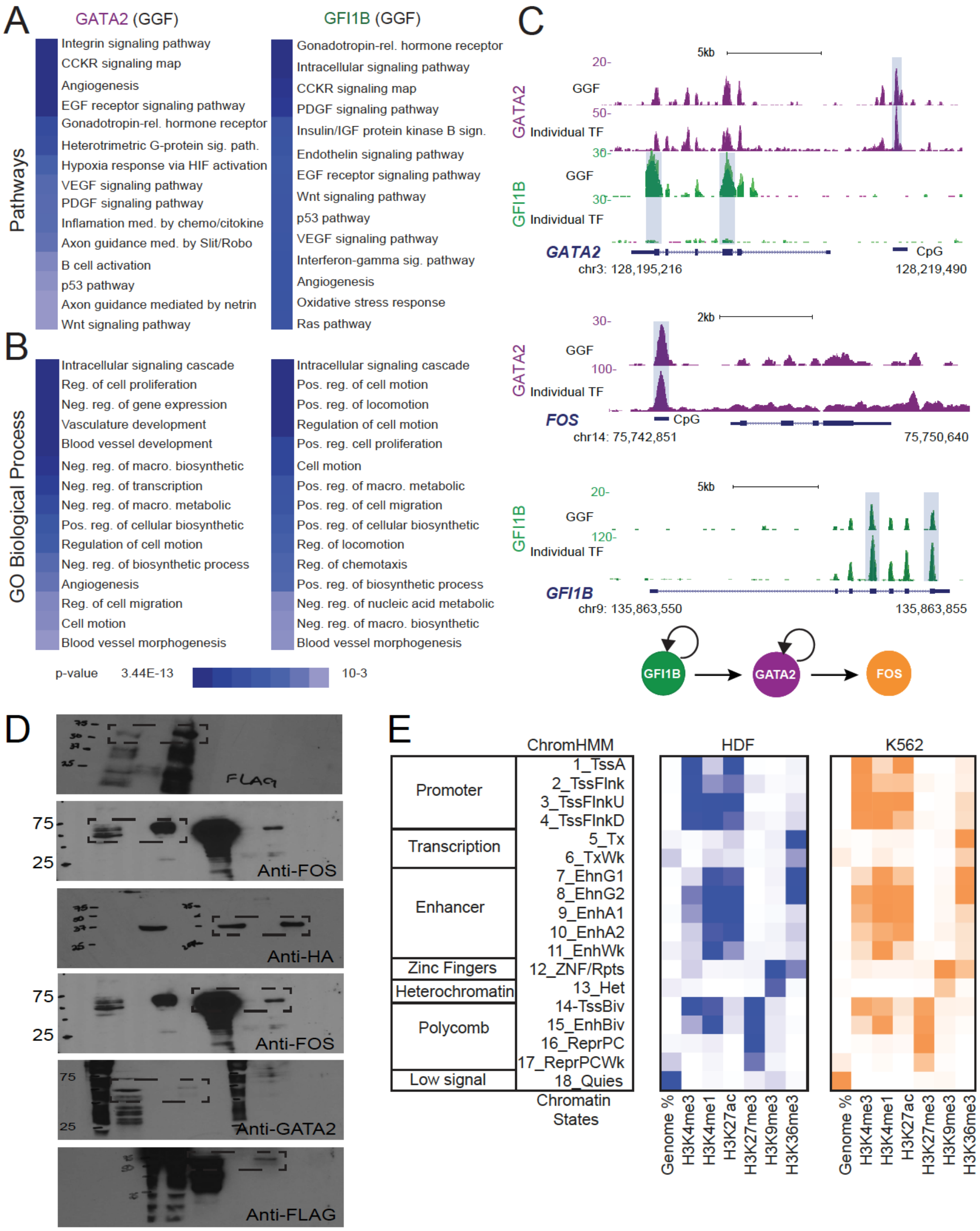

Figure S6. Gene ontology, pathway enrichment and regulatory interactions between GATA2, GFI1B and FOS, Related to Figure 5 and 6. (**A**) Panther pathway enrichment analysis of genes bound by GATA2 (left panel) and GFI1B (right panel) in HDFs transduced with GATA2, GFI1B and FOS (GGF). (**B**) Biological processes GO terms enriched for GATA2 (left panel) and GFI1B (right panel) bound genes in HDF transduced with GGF. (**C**) GATA2 and GFI1B occupancy profile at the *GATA2*, *FOS* and *GFI1B loci*. The scheme summarizes the regulatory interactions between the three transcription factors. (**D**) Uncropped images of immunoblots displayed in Figure 5. Dashed boxes indicate areas that were cropped. (**E**) Rows represent chromatin states according to ChromHMM annotation. TssA (active promoters), TssFlnk (flanking promoters), TssFlnkU (flanking upstream promoters), TssFlnkD (flanking downstream promoters), Tx (strong transcription), TxWk (weak transcription), EnhG1/2 (Genic enhancers), EnhA1/2 (active enhances), EnhWk (weak enhancers), ZNF/Rpts (ZNF genes & repeats), Het (Heterochromatin), TssBiv (Bivalent/Poised TSS), EnhBiv (Bivalent Enhancer), ReprPC (Repressed PolyComb), ReprPCWk (Weak Repressed PolyComb) and Quies (Quiescent). Left panels (blue) show the percentage of genome occupancy for chromatin marks in HDFs. Right panels (orange) show the percentage of genome occupancy for chromatin marks in K562 cells.

Table S4. Datasets analyzed in this study, Related to Figure 2 and 6.

	GEO accession	Cell type	Experiment
H3K4me3	GSM733650	HDFs	ChIP-seq
H3K4me1	GSM1003526	HDFs	ChIP-seq
H3K27ac	GSM733662	HDFs	ChIP-seq
H3K27me3	GSM733745	HDFs	ChIP-seq
H3K9me3	GSM1003553	HDFs	ChIP-seq
H3K36me3	GSM733733	HDFs	ChIP-seq
DNase I	GSM736567	HDFs	ChIP-seq
H3K4me3	GSM733680	K562	ChIP-seq
H3K4me1	GSM733692	K562	ChIP-seq
H3K27ac	GSM733656	K562	ChIP-seq
H3K27me3	GSM733658	K562	ChIP-seq
H3K9me3	GSM733776	K562	ChIP-seq
H3K36me3	GSM733714	K562	ChIP-seq
GATA2	GSM935373	K562	ChIP-seq
FOS	GSM935355	K562	ChIP-seq
GFI1B	GSM1278242	Proerythroblast	ChIP-seq
Hemogenic reprogramming	GSE47497	MEFs	mRNA-seq
Human HSCs	GSE29105	UCB HSCs	Microarray
